# Macrophage restriction in *Drosophila* wounds and tumors via a matrix degradation–moderating protease inhibitor

**DOI:** 10.1126/sciadv.aec5131

**Published:** 2026-09-25

**Authors:** Yoshiki Sakai, Kavya Adiga, Matin Nawabi, Sofia Mendez-Lopez, Tsai-Ching Hsi, David Bilder

**Affiliations:** Department of Molecular and Cell Biology, University of California-Berkeley, Berkeley, CA 94720, USA.

## Abstract

Breaches of epithelial homeostasis trigger an inflammatory response. Not only initiation but also negative regulation of the response is critical, as autoinflammation can cause tissue damage and chronic disease. Epithelial breaches can be signaled by damage-associated molecular patterns (DAMPs), including basement membrane (BM) degradation, that attract inflammatory cells. Here, we show that the conserved glycosylphosphatidylinositol-linked thioester-containing protein Tep3 from *Drosophila*, the ortholog of human CD109, limits innate immune cell attachment to damaged and transformed epithelia. Tep3 is induced in wounds alongside the matrix metalloprotease 1 (MMP1). Tep3 inhibits MMP1 proteolytic activity, reducing production of a BM DAMP that triggers macrophage association. A *Drosophila* tumor up-regulates Tep3 to limit an MMP1- and macrophage-dependent antitumor immune response, thus accelerating progression and host death. Hence, fly tumors can exploit a physiological anti-inflammatory axis to pathologically limit their immune restriction.

## INTRODUCTION

Macrophages play central roles in maintaining tissue homeostasis throughout animal species. Septic and sterile wounds create signals—pathogen- or damage-associated molecular patterns (PAMPs or DAMPs, respectively)—that rapidly recruit macrophages; these and other innate immune cells mediate the initial defense and repair response (*1*). However, this response must be limited to prevent autoinflammatory damage, which is a pathogenic factor in many human diseases (*2*). The resolution of inflammation is much less understood than initiation but involves both the cessation of DAMP and PAMP signals as well as active anti-inflammatory mechanisms.

The macrophage response to wounds bears a fascinating but still unclear relationship to the innate immune response to cancer. In mammalian tumor biology, macrophages play a complex role. Most studies have examined established tumors, where macrophage infiltration is generally thought to be immunosuppressive by adoption of an M2-like phenotype and by promoting efferocytosis, reducing inflammatory cues (*3*, *4*). By contrast, there are fewer studies of early tumors where one might expect the M1-like macrophage presence to increase inflammation and restrain tumor progression. Overall, much remains unknown about such roles in innate tumor immunity, which is a promising frontier for cancer immunotherapy (*5*, *6*).

*Drosophila* has emerged as an interesting model for studying conserved aspects of macrophage biology and the inflammatory response that involves them. Most of fly blood cells are called plasmatocytes, which show differentiation via myeloid-like transcription factors, immune-responsive signaling pathways, and phagocytic receptors that are conserved with vertebrate macrophages (*7*, *8*). These features are sufficiently analogous that plasmatocytes are often referred to as macrophages, as we will do here. Attraction of macrophages to wounds has been extensively studied in the fly embryo, where the main signal involves reactive oxygen species (ROS) (*9*). In larvae, the mechanisms are thought to be different: Circulating macrophages do not chemotax through the larger hemocoel but instead adhere to wound sites through capture when they are in proximity to DAMP cues (*10*). When wound closure is complete, the macrophages disperse, ending the inflammatory response.

Notably, macrophages are also attracted to tumors that form in fly larvae. Fly tumors share a number of hallmarks of mammalian cancer and have become rich systems for studying tumor-host interactions that are conserved between mammals and flies, including lethality-driving syndromes such as cachexia and coagulopathy (*11*–*13*). Macrophages specifically associate with larval fly tumors, with functional consequences that vary depending on the system, including both tumor-promoting and tumor-suppressive outcomes (*11*, *13*–*25*). Excitingly, these data reveal that invertebrate as well as vertebrate tumors elicit an immune response involving innate inflammatory cells.

While much has been learned about fly inflammation, numerous questions remain about the mechanisms that initiate and limit the cellular immune response, especially beyond the embryo. Here, we describe a conserved protease inhibitor that negatively regulates macrophage association with disrupted larval and adult tissues. This molecule, thioester-containing protein 3 (Tep3), is up-regulated with and antagonizes a matrix metalloprotease (MMP) that produces a basement membrane (BM)–dependent DAMP, modulating macrophage association to tumors and wounds with functional consequences for animal homeostasis.

## RESULTS

### An adult *Drosophila* cancer model shows antitumor cellular immunity

**Fig. 1.**
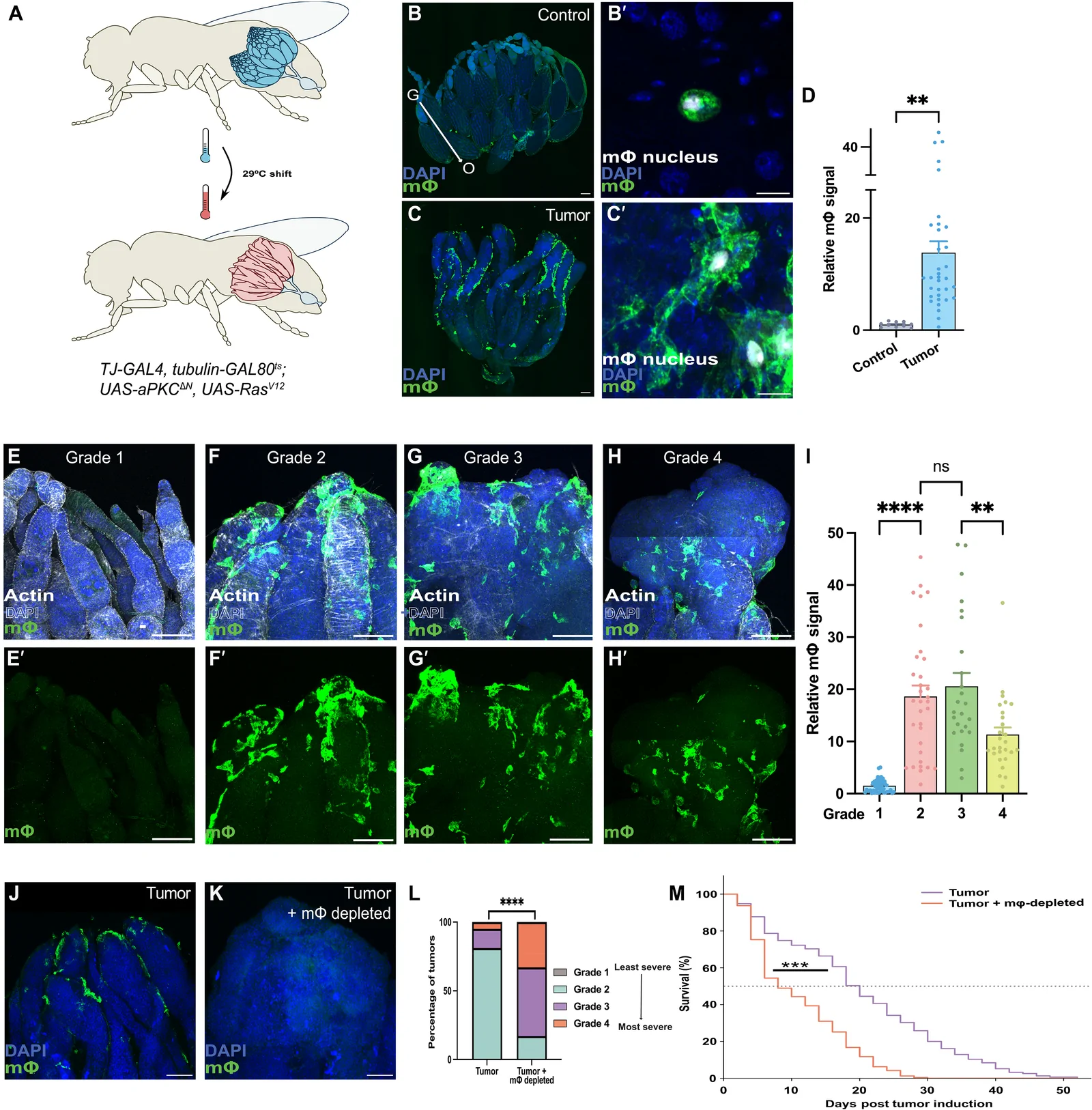
Adult macrophages recognize tumors and slow their progression. (**A**) Diagram of OC tumor model: At 29°C, GAL4 activates transcription of oncogenic aPKC^ΔN^ and Ras^V12^ proteins in the ovarian follicle epithelium. (**B**) WT ovary, oriented as indicated from anterior [germarium (G)] to posterior [oviduct (O)]. Macrophages are detected by anti-NimC1 (green). OC tumors (**C**) exhibit increased macrophages quantitated in (**D**) (*n* values: control = 10; tumor = 34). Control macrophages (also detected by *Hml^P2A^-LHG>NLSmScarlet3* white nuclei) are round (B′), while TAMs display a spread morphology that is associated with macrophage activation (C′). (**E** to **H**) TAMs are not found on grade 1 tumors but start to bind at grade 2 and continue through grades 3 and 4, quantitated in (**I**) (*n* values: grade 1 = 45; grade 2 = 33; grade 3 = 25; grade 4 = 27). (**J** and **K**) Tumor-bearing flies with no macrophages show faster tumor progression and accelerated host death compared to tumor-bearing flies with macrophages, quantitated in (**L**) and (**M**) [*n* values (L): tumor = 58; tumor + no macrophages = 24; (M): tumor = 155; tumor + no macrophages = 239]. Scale bars, 100 μm [(B), (C), (E) to (H), (J), and (K)] and 10 μm [(B′) and (C′)]; unpaired *t* test (D), one-way ANOVA (I), Mann-Whitney *U* test (L), and log-rank test (M) used to determine significance; **P < 0.005, ***P < 0.0005, ****P < 0.00005. ns, not significant. Dots in (D) and (I) represent individual flies.

A cellular immune response to fly tumors has been demonstrated in larval models and transplants but not in autochthonous adult models. We analyzed a genetically engineered fly model that creates an ovarian carcinoma (OC) through expression of the oncogenes *Ras*^*V12*^ and *aPKC*^*act*^ in the follicle cell (ovarian) epithelium, specifically induced in adults via temperature shift (Fig. 1A) (*26*). This model has documented grades of tumor progression, from the appearance of initial transformation at grade 1 to severe grade 4 tumors (fig. S1A), and displays several tumor-host interactions conserved with human patients. We analyzed fly macrophages using an antibody against NimC1, a phagocytic receptor found on most of these cells. In wild-type (WT) ovaries, only a few macrophages are found; these are located close to the germarium and display a compact, rounded morphology (Fig. 1, B and B′); macrophages are also seen on the posterior oviduct. By contrast, tumorous ovaries show a marked increase in macrophages, which are primarily associated with the transformed cells at the ovary anterior (Fig. 1, C and D). Tumor-associated macrophages (TAMs) have a spread morphology with multiple cellular extensions, a phenotype previously termed “macrophage activation” (Fig. 1C′) (*27*). We examined the kinetics of TAMs with respect to OC progression (Fig. 1, E to I). TAMs were first seen on grade 2 tumors, as evidenced by a 20-fold increase in NimC1 reactivity. Elevated macrophage numbers continued in grade 3 and reduced slightly in grade 4 as compared to grade 3 tumors.

To test a functional role in the OC model, we generated flies in which macrophages were ablated through GAL4-driven expression of apoptosis inducers, specifically in adults. OC tumors were driven by LexA-based expression of *Ras*^*V12*^ and *aPKC*^*act*^ in follicle cells, which produces tumors similar to those driven by GAL4. The ablation system used eliminates most macrophages (*28*), and no TAMs were seen in these flies (Fig. 1K). Macrophage-depleted flies showed strongly accelerated tumor progression, with a ∼6.5-fold increase in grade 4 tumors and a nearly fourfold increase in grade 3 tumors at day 15 after tumor induction (ATI), compared to macrophage-replete control flies (Fig. 1, J to L). Macrophage depletion caused a slight reduction in tumor apoptosis (fig. S1, B to D). Macrophage depletion accelerated the death of tumor-bearing flies, although as previously shown, it also reduced life span in non–tumor-bearing flies (Fig. 1M and fig. S1E) (*17*, *29*–*32*). Therefore, the OC model induces a cellular immune response in the adult that has a net antitumor activity.

### Ovarian tumors express a protein that restricts TAMs

**Fig. 2.**
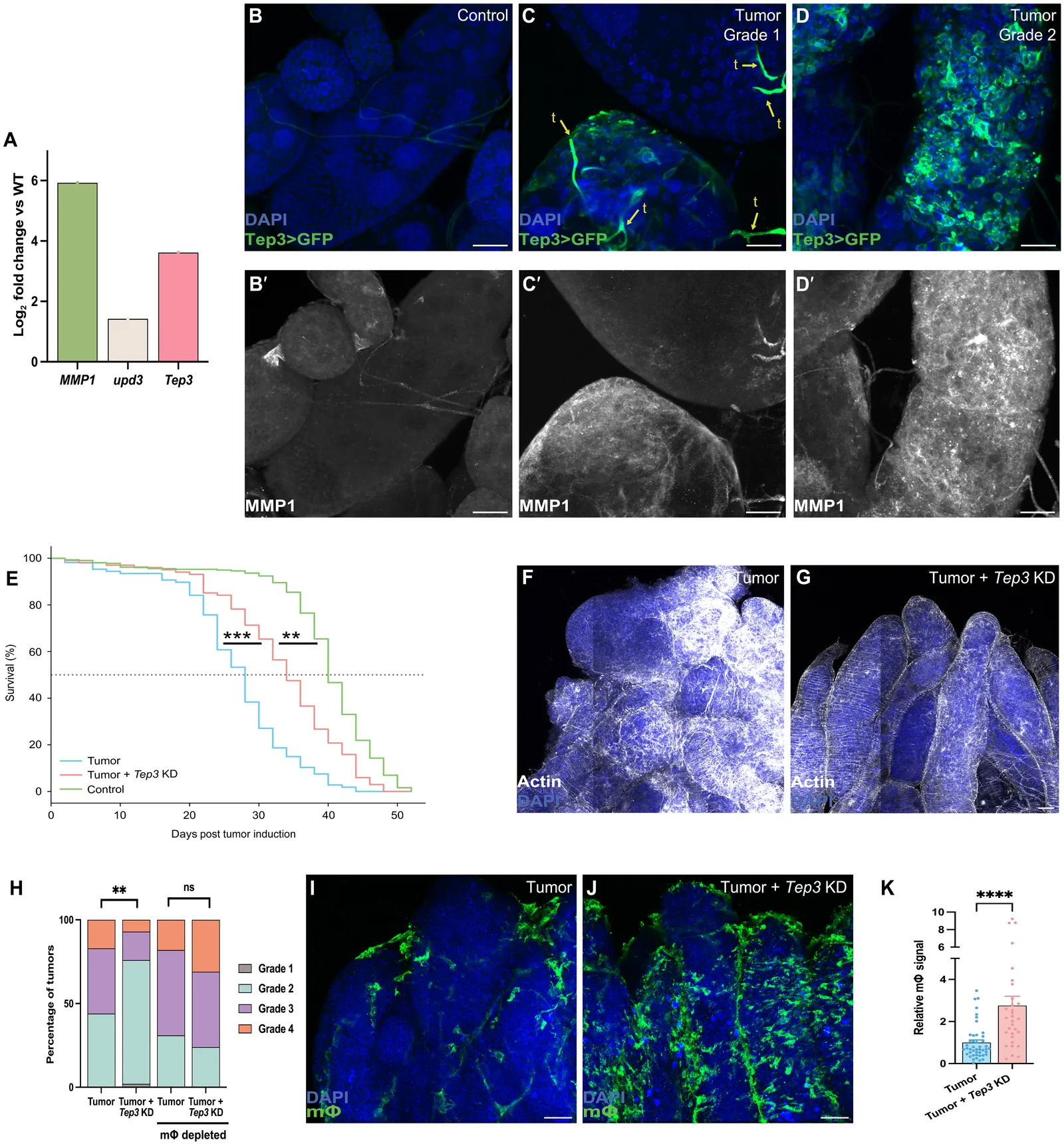
Tumor-produced Tep3 limits macrophage association. (**A**) Elevation of oncokine transcripts in the OC model. OC transcriptome is from Hsi *et al.* (*26*). OC tumors up-regulate *Tep3* alongside expected genes such as pro-invasion *MMP1* and pro-inflammation *upd3.* (**B** to **D**) *Tep3* levels are increased in tumors compared to control ovaries (Tep3>GFP: *Tep3-GAL4>mCD8GFP*). *Tep3* is also expressed in ovary-associated trachea (t). [(B′) to (D′)] *Tep3* expression is correlated with MMP1 antibody staining. Tumors with *Tep3* knockdown (KD) display increased host life span (**E**) and slower tumor progression (**F** and **G**) [DAPI (blue) and actin (white)], quantitated in (**H**) [*n* values: (E) tumor = 107; tumor + *Tep3* KD = 87; (H) tumor = 36; tumor + *Tep3* KD = 53; mφ-depleted tumor = 39; mφ-depleted tumor + *Tep3* KD = 71]. Macrophage-depleted flies show no significant difference in tumor progression between WT and *T*e*p3 KD* tumors, indicating that the Tep3 effect on tumor progression is macrophage-dependent. (**I** and **J**) Tumors with *Tep3* KD exhibit increased TAMs (anti-NimC1, green), quantitated in (**K**) (*n* values: tumor = 45; tumor + *Tep3 KD* = 31). Scale bars, 20 μm [(B), (B′), (C), (C′), (D), and (D′)] and 50 μm [(F), (G), (I), and (J)]; unpaired t-test (K), Mann-Whitney *U* test (H), and log-rank test (E) used to determine significance; **P < 0.005, ***P < 0.0005, ****P < 0.00005. Dots in (K) represent individual samples.

To explore mechanisms that could regulate macrophages in OC flies, we tested oncokines: secreted factors that are up-regulated in the tumor and could mediate interactions with nontumor cells. Among a list of oncokines from RNA sequencing of OC tissue, including those previously validated to mediate tumor-host communication (*11*, *26*, *33*, *34*), we focused on Tep3. *Tep3* is up-regulated ∼12-fold in the OC transcriptome (Fig. 2A) (*26*). We confirmed up-regulation by examining a *Tep3-GAL4* transcriptional reporter in OC tumors. *Tep3* expression was not evident in control ovaries but was first elevated in subsets of transformed cells within grade 1 tumors, with expression increased in tumors of grades 2 and higher (Fig. 2, B to D).

For functional tests, we depleted Tep3 using an effective RNA interference (RNAi) construct (fig. S2, A and B), specifically in the follicle cells alongside the two oncogenes. Tep3 depletion in tumors induced a strong extension of the life span of tumor-bearing flies (Fig. 2E). Analysis of tumor grade revealed that Tep3-depleted tumors progressed more slowly than those depleted for a control RNAi construct, consistent with the life-span extension (Fig. 2, F to H). Extension was also seen with a second independent RNAi construct (fig. S2C). Thus, tumor-produced Tep3 accelerates tumor progression and host death.

Given that depleting either macrophages or Tep3 leads to opposite effects on tumor progression and life span, we next analyzed TAMs. Notably, Tep3-depleted tumors displayed nearly threefold more TAMs than control tumors (Fig. 2, I to K). The difference was independent of tumor grade: that is, both grade 2 and grade 3 Tep3-depleted tumors contained more TAMs than control counterparts. Tep3 depletion had no impact on macrophage numbers on otherwise WT ovaries (fig. S2D). This suggested that Tep3 is produced by the tumor to limit macrophage association. We then depleted Tep3 in OC tumors while eliminating macrophages with apoptotic inducers using LexA-driven expression. When macrophages were killed, Tep3 depletion lost its ability to slow tumor progression; there was no significant difference in tumor grades between Tep3-expressing and Tep3-depleted tumors (Fig. 2H and fig. S2, E and F). These results suggest that Tep3 acts via macrophages to limit the host antitumor immune response.

### BM digestion cues macrophages to attach to the tumor

How could Tep3 negatively regulate macrophage association with OC tumors to accelerate progression and host death? Both invertebrate and vertebrate phyla contain multiple thioester-containing protein (TEP) family members. Perhaps the best known is vertebrate α-2-macroglobulin (a2M), which is secreted to regulate inflammation and immunity by directly inhibiting plasmatic proteases (*35*). Evolutionary duplication of a2M is thought to have given rise to the soluble complement proteins C3, C4, and C5; C3 and C4 use the reactive thioester to bind covalently to pathogenic cells, while C5 has lost the reactive site (*36*). The *Drosophila* genome encodes four TEP family members that are phylogenetically closer to a2M than to the complement proteins. Insect TEPs have been previously investigated for immune roles, including acting as opsonins, being protease inhibitors, and participating in complement pathways (*37*, *38*). Tep3 is perhaps the least studied and may not be induced by microbial challenge like Tep1, Tep2, and Tep4 (*39*–*42*). Tep3 is also the sole fly TEP to have a predicted glycosylphosphatidylinositol (GPI) modification site. GPI linkages anchor secreted proteins in the outer leaflet of the plasma membrane, suggesting a local rather than systemic role. Although no Tep3-specific antibody is available, expression of a tagged transgene confirmed Tep3 membrane enrichment (fig. S2A). The predicted human ortholog of Tep3 is CD109, which is also the sole GPI-anchored TEP in our species (*43*).

**Fig. 3.**
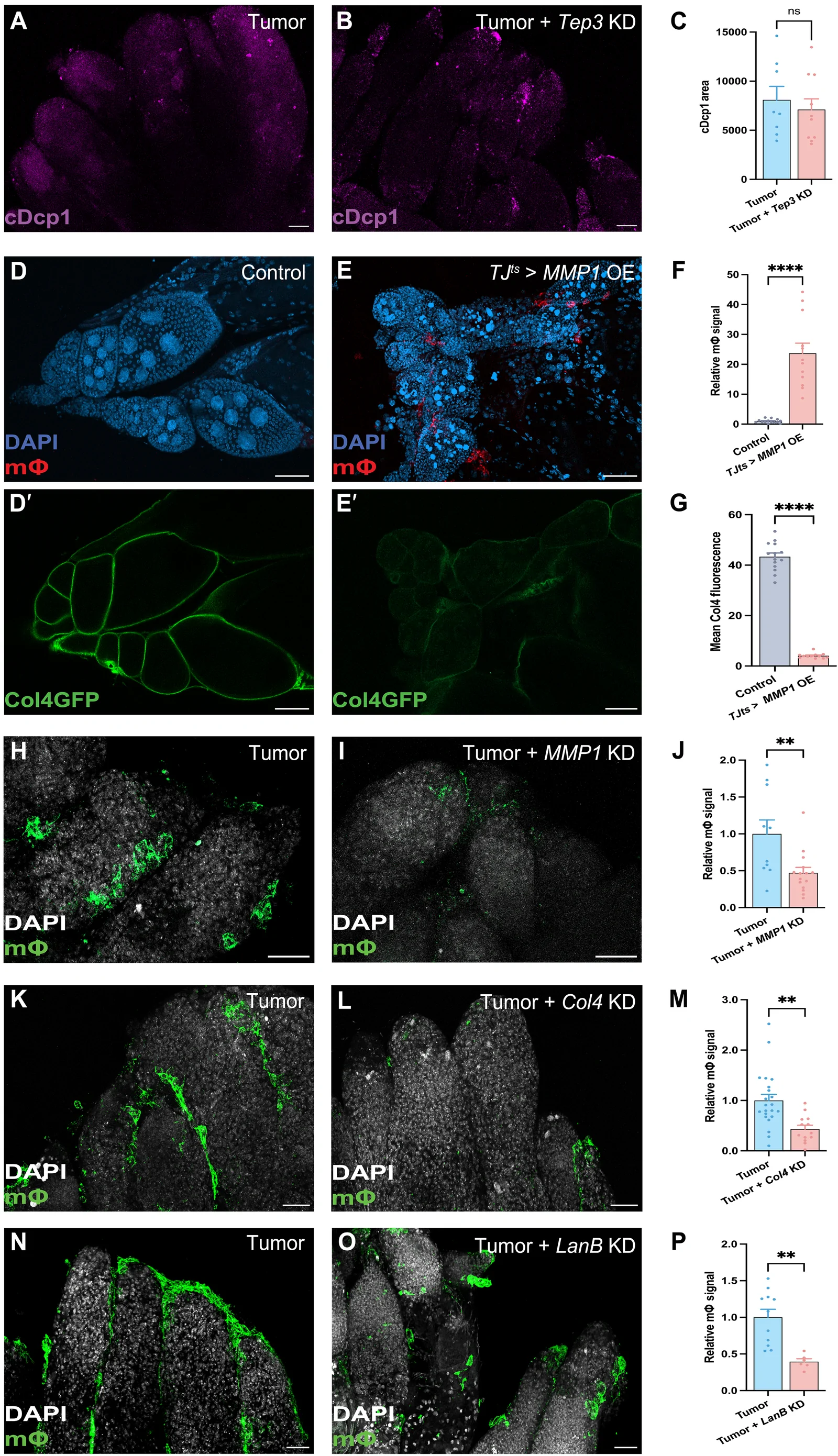
Macrophage association with tumor involves BM breakdown. (**A** and **B**) *Tep3* KD in tumors does not affect apoptosis at day 10 ATI, indicated by anti-cDcp1 staining (magenta), quantitated in (**C**) (*n* values: tumor = 29; tumor + *Tep3* KD = 16). (**D** and **E**) *MMP1* overexpression in a WT ovary is sufficient to recruit macrophages (anti-NimC1, red) and reduce BM levels [(D′ and E′); vkgGFP (Col4GFP) in green, single confocal slice], quantitated in (**F**) and (**G**) [*n* values: (F) control = 14; *MMP1* overexpression (OE) = 12; (G) control = 14; *MMP1* OE = 12]. (**H** and **I**) Tumors with *MMP1* KD exhibit decreased TAMs (anti-NimC1, green), quantitated in (**J**) (*n* values: tumor = 10; tumor + *MMP1* KD = 15). Depleting Col4 (**K** and **L**) or Laminin (**N** and **O**) in OC tumors reduced TAMs (anti-NimC1, green), quantitated in (**M**) and (**P**) [*n* values: (M) tumor = 22; tumor + *Col4* KD = 13; (P) tumor = 11; tumor + *lanB* KD = 6]. Scale bars, 50 μm; unpaired *t* test [(C), (F), (G), (J), (M), and (P)] used to determine significance; **P < 0.005, ****P < 0.00005. Dots in (C), (F), (G), (J), (M), and (P) represent individual samples.

A familiar paradigm for macrophage recognition of wounded or transformed tissue involves DAMPs that signal the presence of dying or unwanted cells, triggering their removal (*44*, *45*). ROS is one characterized DAMP to which both fly and vertebrate macrophages respond, while dying cells can release a variety of components that have DAMP activity. OC tumors show apoptosis not seen in WT ovaries, but we found no difference in apoptosis between OC tumors with and without Tep3 depletion at day 10 ATI (Fig. 3, A to C). Inducing cell death in nontumorous ovaries was not sufficient to attract macrophages, and blocking cell death in OC tumors through overexpression of DIAP1 or p35 also had no effect on TAM numbers (fig. S3, A to E). The same was true for OC tumors in which ROS was reduced by depletion of dual oxidase (fig. S3, F to I).

In fly larvae, breaches of the BM due to either mechanical disruption or enzymatic degradation result in macrophage attachment (*15*, *16*, *19*, *46*). To our knowledge, past experiments do not distinguish whether macrophages recognize the absence of BM components on tissue per se or, alternatively, the activity of BM-degrading enzymes such as MMPs. Given that follicle cells produce most of their own BM (*47*), we first depleted the BM components Collagen IV (Col4) or Laminin from otherwise WT ovaries using effective RNAi constructs (fig. S4, G to J) and found that both caused a minor (approximately twofold) increase in macrophages, predominantly in the “companion” population at the germarium (fig. S4, A to F) (*47*). We then induced BM degradation through transgenic expression of either of the two MMPs encoded in the fly genome, named MMP1 and MMP2. While ovaries overexpressing MMP2 could not be recovered, overexpression of MMP1 in otherwise WT follicle cells was sufficient to reduce BM levels and cause a strong 20-fold increase in macrophage attachment (Fig. 3, D to G, and fig. S4, K to M).

We next analyzed the OC model. Previous transcriptome data showed that *MMP1* is up-regulated in these tumors (Fig. 2A) (*26*), and BM levels are reduced (fig. S4, S to U). To functionally test a role for MMP1, we depleted it from OC tissue. At day 10 ATI, when TAMs are first seen on control tumors, MMP1-depleted tumors showed decreased TAM numbers, with no significant change in tumor grade compared to control tumors (Fig. 3, H to J, and fig. S4N). When Col4 or Laminin was depleted in OC, decreased TAMs were also seen in grade-matched comparisons (Fig. 3, K to P, and fig. S4, O to R). Together, these data indicate that tumor MMP1 digests BM to release the relevant epithelial DAMP for macrophage association.

### Tep3 inhibits MMP1 activity to modulate macrophage association

**Fig. 4.**
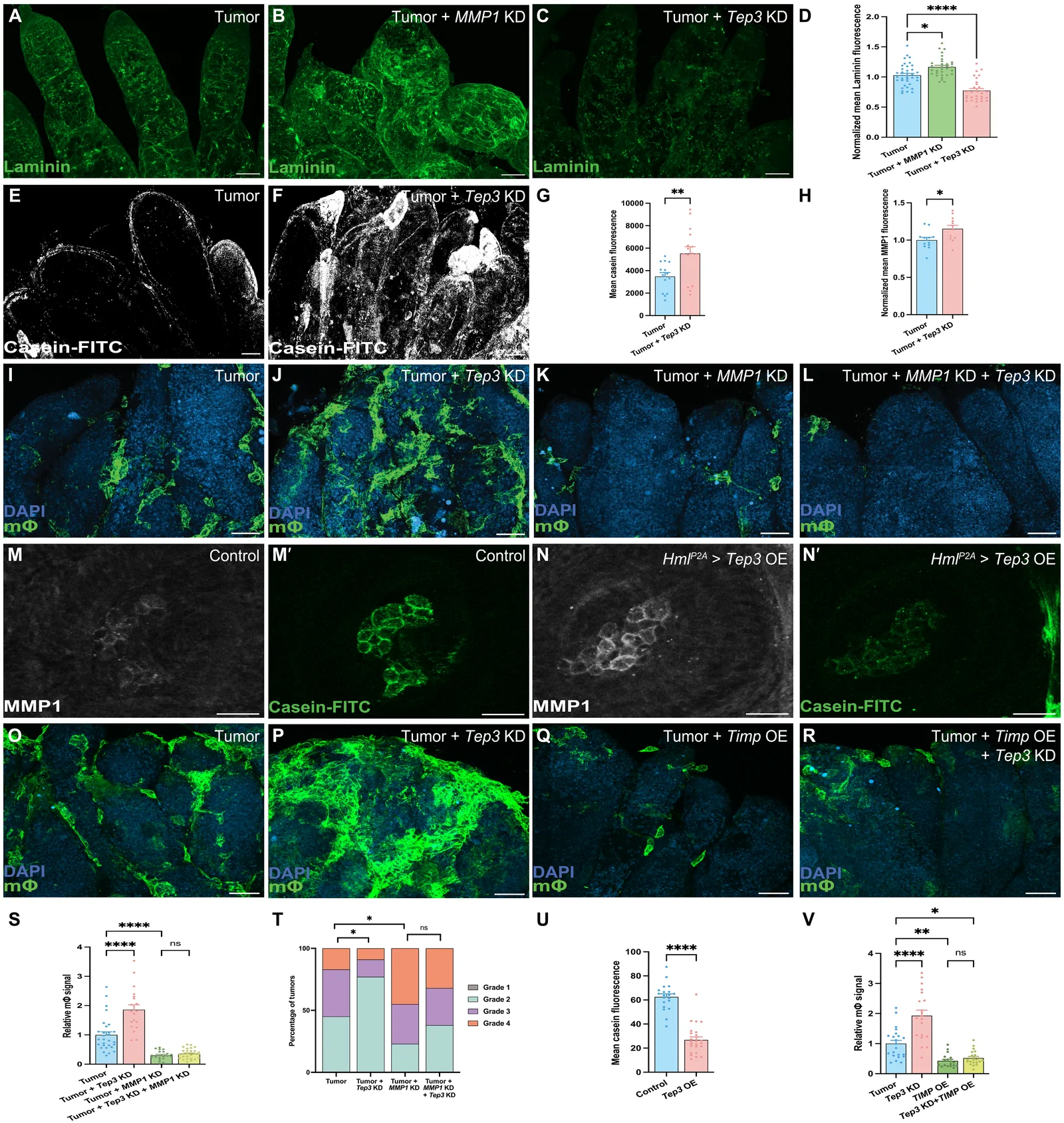
Tep3 inhibits MMP1 activity to modulate immune recognition of tumor. Compared to control tumors (**A**), tumors with *MMP1* KD show increased BM (anti-Laminin, green) (**B**), while tumors with *Tep3* KD display decreased BM (**C**), quantitated in (**D**) (*n* values: tumor = 37; tumor + MMP1 KD = 32; tumor + Tep3 KD = 30). (**E** and **F**) Tumors with *Tep3* KD display increased MMP1 activity (casein-FITC fluorescence, white), quantitated in (**G**) (*n* values: tumor = 14; tumor + Tep3 KD = 15). (**H**) *Tep3* KD tumors have slightly increased MMP1 levels (*n* values: tumor = 12; tumor + Tep3 KD = 12). (**I** to **L**) Tep3 function in tumors depends on MMP1, as the TAM increase (anti-NimC1, green) in *Tep3* KD tumors compared to control tumors [(I) and (J)] is lost with *MMP1 Tep3* double KD, which is similar to *MMP1* KD alone [(K) and (L)], quantitated in (**S**) (*n* values: tumor = 29; tumor + *Tep3* KD = 19; tumor + *MMP1* KD = 20; tumor + *Tep3* KD + *MMP1* KD = 28). Slower tumor progression caused by *Tep3* KD is also lost upon *MMP1 Tep3* double KD (**T**) (*n* values: tumor = 40; tumor + *Tep3* KD = 44; tumor + *MMP1* KD = 44; tumor + *Tep3* KD + *MMP1* KD = 53). (**M** and **N**) WT eye-antennal disc macrophages express MMP1 (white) and display casein cleavage (green); *Tep3* overexpression in macrophages reduces MMP1 activity [quantitated in (**U**)] but not protein levels (*n* values: control = 20; Tep3 OE = 24). (**O** to **R**) Tep3 function in limiting TAMs [(O) and (P)] can be rescued by provision of TIMP [(Q) and (R)], quantitated in (**V**). Scale bars, 50 μm, except (M) and (N) = 20 μm; unpaired *t* test [(G), (H), and (U)], Kruskal-Wallis test (T), and one-way ANOVA [(D), (S), and (V)] used to determine significance; *P < 0.05, **P < 0.005, ****P < 0.00005. Dots represent individual samples.

The activity of TEP family proteins as protease inhibitors suggested a hypothesis that Tep3 inhibits MMP1 activity to regulate TAMs in the OC model. Immunostaining for MMP1 protein showed elevation first in some cells of grade 1 tumors (Fig. 2C). These MMP1-positive cells often though not always coexpressed the *Tep3* transcriptional reporter. At later stages, MMP1 became more widespread throughout the tumor, as did *Tep3* (Fig. 2D). Notably, BM levels were significantly lower in Tep3-depleted tumors compared to control grade-matched tumors, whereas MMP1 depletion increased BM levels (Fig. 4, A to D), suggesting the hypothesis that Tep3 might oppose MMP1-mediated BM destruction.

Given that TEP proteins inhibit protease activity rather than protease levels, we turned to a fluorometric assay. MMP1 protease activity can be detected by liberation of fluorescein isothiocyanate (FITC) otherwise quenched by casein, a demonstrated MMP1 substrate (*48*). When incubated with casein-FITC, tumors revealed FITC dequenching, while WT ovaries did not, correlating with MMP1 immunostaining (fig. S5, A to C). We then used the casein-FITC assay on Tep3-depleted tumors. Fluorescence quantitation showed a clear increase in grade-matched tumors compared to tumors depleted with a control RNAi (Fig. 4, E to G). We examined whether higher MMP1 enzymatic activity was due to MMP1 protein up-regulation and found only a slight increase in Tep3-depleted versus control tumors (Fig. 4H and fig. S4, V and W).

An implication of the hypothesis that tumor-produced Tep3 restricts MMP1 activity is that MMP1 produces the DAMP sensed by OC TAMs. We therefore examined OC tumors that were depleted simultaneously for both MMP1 and Tep3. These tumors showed no significant difference in TAMs compared to MMP1 depletion alone (Fig. 4, I to L and S). By day 20 ATI, MMP1-depleted tumors showed faster tumor progression than control tumors, and the same accelerated progression was seen in MMP1 Tep3 double-depleted tumors (Fig. 4T). Thus, up-regulated MMP1 increases TAM numbers, and Tep3 requires MMP1 to limit this phenotype.

To further test the relationship between Tep3 and MMP1, we sought an independent site of native, nonpathological MMP1 expression. We noticed that macrophages associated with the eye-antennal imaginal disc of WT L3 larvae expressed MMP1 (Fig. 4M and fig. S5D) and, as expected, consistently liberated casein-FITC (Fig. 4M′). Driving transgenic expression of Tep3 in hemocytes reduced casein cleavage in the fluorometric assay (Fig. 4, N and U). There was no reduction in MMP1 levels; MMP1 levels were slightly elevated in the presence of Tep3 overexpression for unknown reasons.

Last, if Tep3 inhibits macrophage association by limiting BM damage, its role should be replaceable with other MMP1 inhibitors. The tissue inhibitor of metalloprotease (TIMP) family of conserved proteins are well-characterized antagonists of most MMPs (*49*). They are structurally and phylogenetically unrelated to TEPs and inhibit MMPs by binding directly to the catalytic cleft. *Drosophila* has a single TIMP that antagonizes MMP1 as well as MMP2 (*50*). When overexpressed in tumors, TIMP was sufficient to reverse the excessive macrophage association seen when Tep3 was depleted (Fig. 4, O to R and V), consistent with the proposed hypothesis of Tep3 action.

### Wound-induced Tep3 inhibits MMP1 and macrophages in physiological contexts

**Fig. 5.**
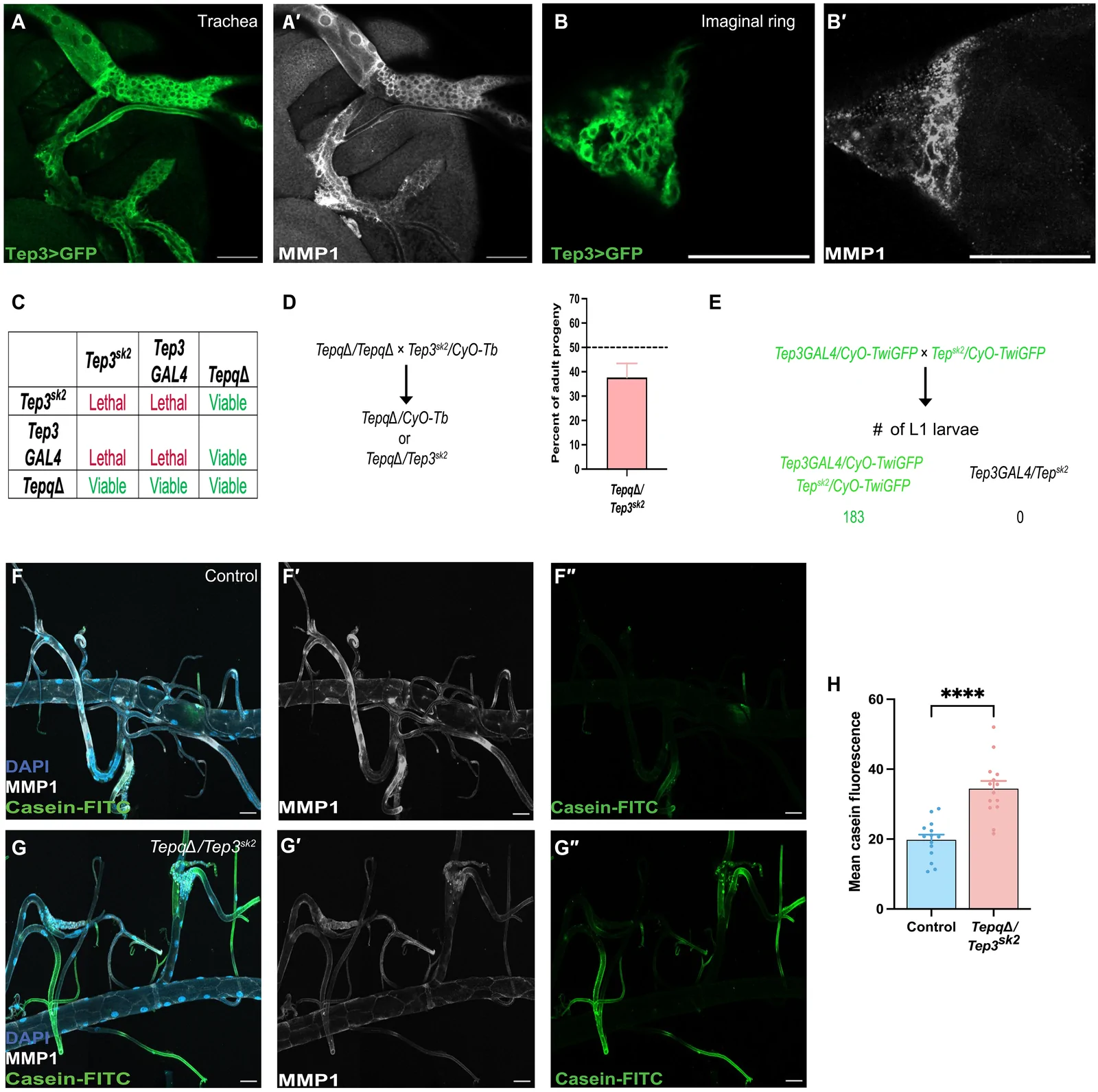
Tep3 antagonizes MMP1 during normal development. *Tep3GAL4>mCD8GFP* (green) is expressed in L3 trachea (**A**) and salivary gland imaginal ring (**B**); at both sites, it colocalizes with expression of MMP1 (white). (**C**) The complementation matrix shows that *Tep3* is an essential gene and that *Tepq*^Δ^ is hypomorphic. (**D**) *Tepq*^Δ^/*Tep3^sk2^* adults eclose at a sub-Mendelian ratio (*P* < 0.0001). Data represent the combined progeny from two independent crosses (107 *Tepq*^Δ^/*Tep3^sk2^* among 284 total progeny). The dashed line indicates the expected Mendelian ratio (50%). Error bars indicate the 95% confidence interval. (**E**) *Tep3GAL4/Tep3^sk2^* animals show an embryonic lethal phenotype. No transheterozygous embryos hatch from a *Tep3GAL4/CyO-TwiGFP* × *Tep3^sk2^/CyO-TwiGFP* cross. (**F** and **G**) *Tepq*^Δ^/*Tep3^sk2^* larvae display elevated casein-FITC cleavage (green) in MMP1-expressing (white) trachea, quantitated in (**H**) (*n* values: control = 14; *Tepq*^Δ^/*Tep3^sk2^* = 14). Scale bars, 50 μm; two-sided exact binomial test (D) and unpaired *t* test (H) used to determine significance; ****P < 0.00005. Dots in (H) represent individual samples.

**Fig. 6.**
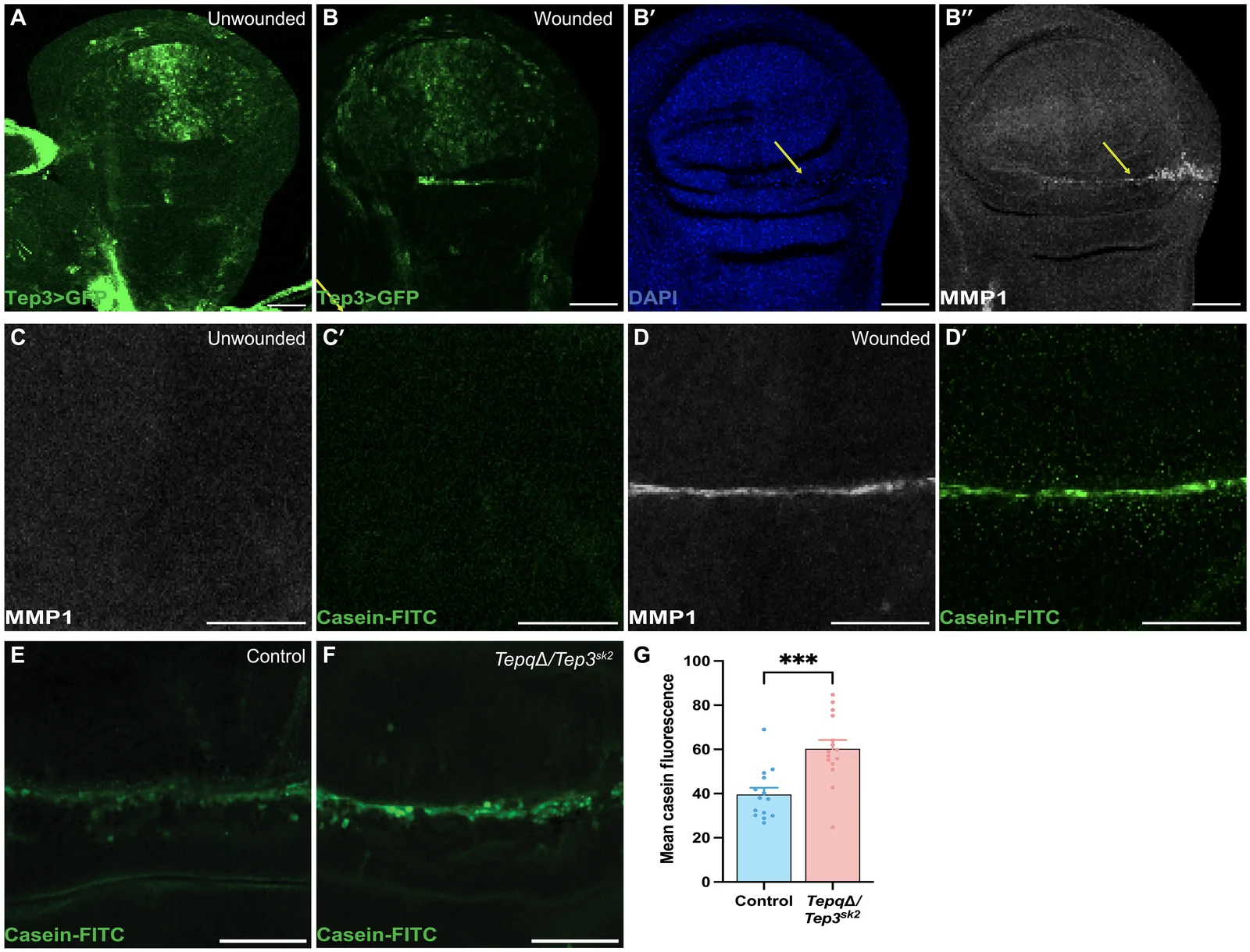
Wound-induced Tep3 restrains MMP1 activity. (**A**) WT larval wing discs express *Tep3* (*Tep3GAL4>mCD8GFP*, green) in the future wing blade. (**B**) Wounded wing discs [marked by a yellow arrow, with pyknotic nuclei revealed by DAPI (blue)] show induction of MMP1 (white) and colocalized *Tep3* (green). Magnified insets are shown in fig. S5E. (**C** and **D**) Wound MMP1 (white) is correlated with increased casein cleavage (green), indicating elevated MMP1 activity. Compared to wounded WT discs (**E**), casein cleavage is elevated when *Tepq*^Δ^/*Tep3^sk2^* discs are wounded (**F**), quantitated in (**G**) (*n* values: control = 14; *Tepq*^Δ^/*Tep3^sk2^* = 15). Scale bars, 50 μm; unpaired *t* test (G) used to determine significance; ***P < 0.0005. Dots in (G) represent individual samples.

Why would a tumor produce both a BM-cleaving MMP and an inhibitor of that enzyme? Given that cancer often activates physiological pathways in pathological contexts, we considered what the normal functions of Tep3 might include. We first examined transcriptional reporters in WT L3 larvae. *Tep3* is expressed in imaginal discs, including the future wing blade, as well as many tracheae and the imaginal ring of the salivary gland (Figs. 5, A and B, and 6A). Notably, the latter two cell types are known to produce MMP1 (*50*, *51*), and partial overlap with *Tep3* expression was confirmed by immunostaining (Fig. 5, A′ and B′). For functional tests, we turned to *Tep3* mutant flies. Prior work suggested that *Tep3* is not required for adult viability (*40*, *41*), but the allele studied (*Tepq*^Δ^) removes only putative *Tep3* 5′ regulatory sequences. However, complementation testing using alleles that interrupt the coding sequence, including CRISPR-generated deletion (*Tep3^sk2^*) and transposon insertion (*Tep3-GAL4^CRIMIC^*), indicates that *Tepq*^Δ^ is a hypomorph and that *Tep3* null animals are embryonically lethal (Fig. 5, C to E). We analyzed hypomorphic *Tepq*^Δ^/*Tep3^sk2^* L3 larvae and found elevated casein-FITC cleavage activity in tracheal tissues, although not the salivary gland (Fig. 5, F to H). These data reveal that Tep3 is an essential gene and a physiological regulator of MMP1 activity.

**Fig. 7.**
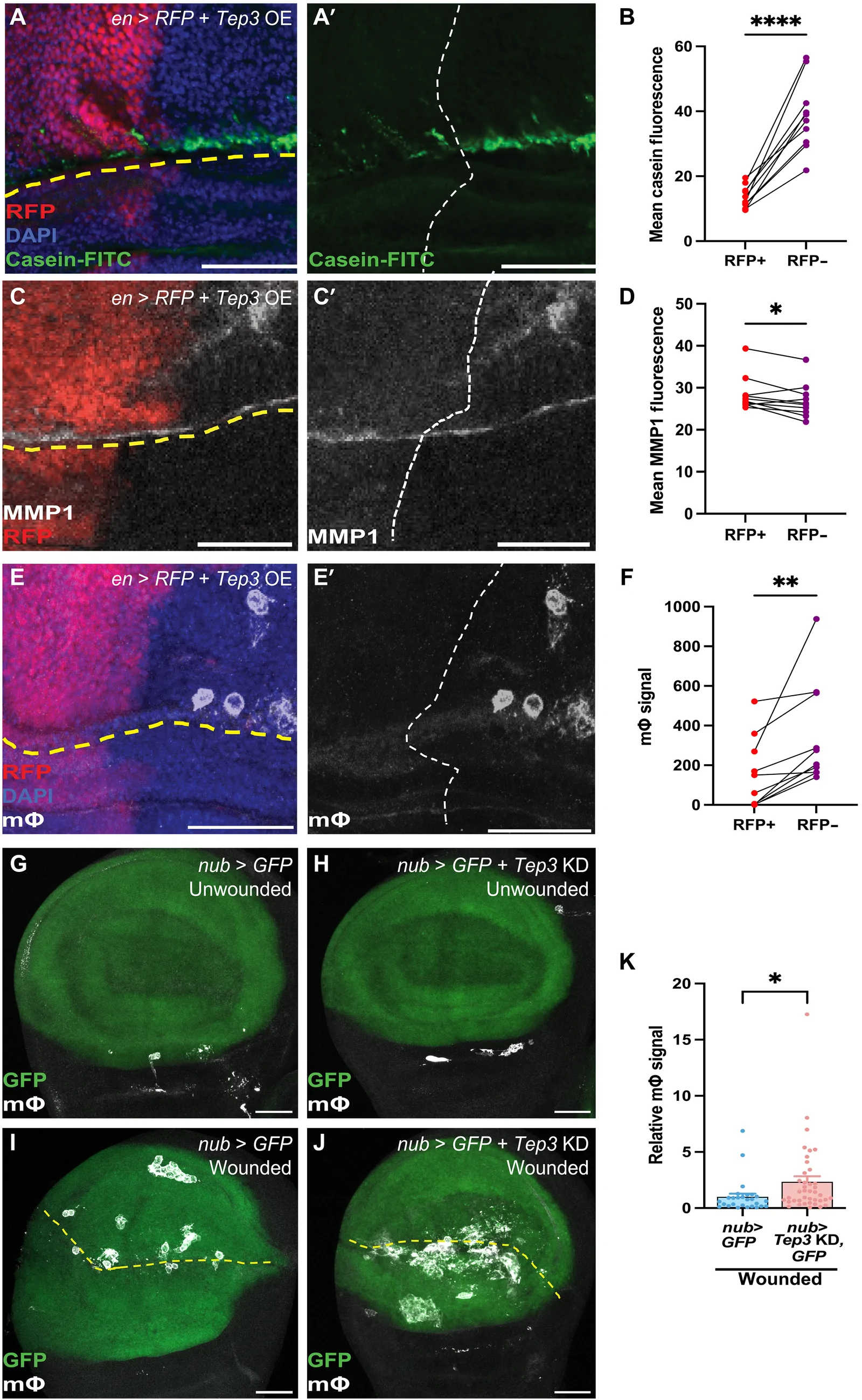
Tep3 mutes the macrophage response to tissue damage. Overexpressing *Tep3* in the anterior compartment of a wounded wing disc specifically reduces MMP1 activity (green) (**A**) in *Tep3-*expressing wound cells (*engrailed*-expressing region in red, wound marked by a yellow line), quantitated in (**B**), without reducing MMP1 levels (white) (**C**), quantitated in (**D**) [*n* values: (B) 10 and (D) 10]. Macrophage association (white) (**E**) is also decreased in the *Tep3-*expressing region, quantitated in (**F**) (*n* values: 10). Tep3 depletion in the wing blade does not alter macrophage association with unwounded discs (**G** and **H**) but increases macrophage association when discs are wounded (**I** and **J**), quantitated in (**K**) (*n* values: *nub>GFP* = 27; *nub>Tep3KD, GFP* = 39). Scale bars, 50 μm; unpaired *t* test (K) and paired *t* test [(B), (D), and (F)] used to determine significance; *P < 0.05, **P < 0.005, ****P *<* 0.00005. Dots in (B), (D), (F), and (K) represent individual samples.

Epithelial wounds share many characteristics with tumors, including disruption of tissue structure and attachment of macrophages (*52*, *53*). To investigate whether Tep3 played a role in tissue repair, we first wounded WT wing discs by physically pinching them, both ex vivo and in vivo. In ex vivo culture, MMP1 was up-regulated at the wound site ∼3 hours after damage, alongside pyknotic nuclei typical of dying cells; the wound site also displayed MMP1 activity revealed by the casein-FITC assay (Fig. 6, B to D, and fig. S5E). MMP1 colocalized here with new wound-induced production of the *Tep3* transcriptional reporter (Fig. 6B and fig. S5E). *Tep3* induction also colocalized with necrotic tissue damage (fig. S5F). When *Tepq*^Δ^/*Tep3^sk2^* hypomorphic wing discs were wounded, casein-FITC cleavage was elevated compared to wounded WT discs (Fig. 6, E to G). By contrast, when Tep3 was overexpressed in the anterior compartment of a wounded disc, it caused a significant reduction of MMP1 activity, with no reduction in MMP1 levels (Fig. 7, A to D).

To determine whether Tep3-regulated MMP1 activity affected wound healing, we attempted to score the regeneration of adult wings (*54*). However, Tep3 depletion alone caused variable defects in wing unfolding, preventing confidence in scoring wound recovery. We instead turned to analyze macrophage recruitment. Consistently, overexpressing Tep3 in the anterior compartment of a wounded disc locally reduced macrophage association (Fig. 7, E and F). Depletion of Tep3 in unwounded discs had no effect on macrophage association (Fig. 7, G and H). As previously reported (*19*), wounded WT discs show a small number of associated macrophages (Fig. 7, I and K). When Tep3 was depleted and discs were wounded, this number increased more than twofold (Fig. 7, J and K). Thus, antagonism of MMP1 and macrophage attachment by Tep3 in tumors reflects a normal function in WT homeostasis.

## DISCUSSION

**Fig. 8.**
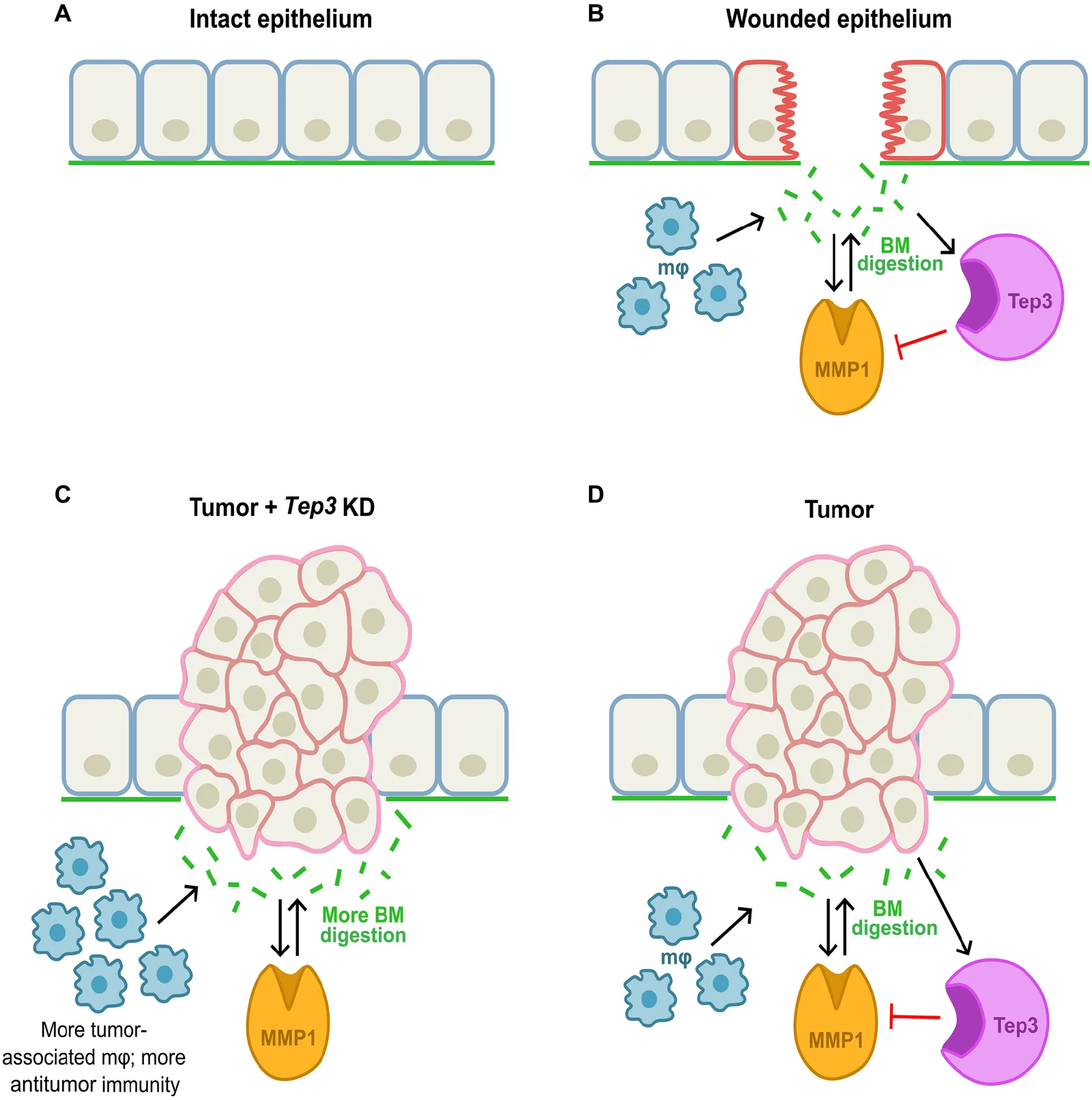
Model for Tep3 activity in wounds and tumors. (**A** and **B**) Disruptions of epithelial integrity in physiological wounds, likely sensed by JNK signaling, trigger transcriptional up-regulation and secretion of MMP1. MMP1 locally remodels BM, promoting a signal that is sensed by macrophages to assist with repair. Wounding also up-regulates Tep3 to limit MMP1 activity and allow resolution of inflammation. (**C** and **D**) Tumors trigger the same damage response, recruiting macrophages that provide antitumor immune activity. In the absence of Tep3, enhanced damage signaling increases macrophage attraction, and consequently, immune restriction of tumors is enhanced.

Mechanisms that regulate immune cell recruitment are central to both physiological and pathological inflammatory responses. We show here that fly Tep3 is a macrophage association-limiting molecule that is induced upon epithelial disruption (Fig. 8). TEPs from many species have immune- or inflammation-related roles, including the mammalian proteins C3/C4 that function in the complement system and a2M, which is released during tissue injury (*35*). Previously proposed functions for insect TEPs include systemic action as opsonins, a2M-like plasmatic protease inhibition, and participation in a complement-related pathway (*38*, *42*, *55*–*57*). While these may be the case for the infection-induced secreted *Drosophila* Tep1, Tep2, and Tep4, they are unlikely to be the case for GPI-linked Tep3. Instead, Tep3 is up-regulated and locally active in both tumors and wounded tissue, where it works to antagonize MMP1, as demonstrated by the MMP1 dependence of examined *Tep3* phenotypes. Like inflammatory MMP1, anti-inflammatory Tep3 induction may be activated by the wound-induced c-Jun N-terminal kinase (JNK) pathway, and the two proteins are coexpressed in a number of (although not all) contexts; inspection of datasets reveals JNK-dependent *Tep3* up-regulation alongside *MMP1* in larval tumors as well as regenerating wounds (*58*, *59*). We suggest that Tep3 expression is normally dynamically tuned to limit MMP1 activity to appropriate levels, preventing excess BM degradation and helping to resolve inflammation.

A direct target of Tep3 inhibitory activity remains unknown. Data are consistent with the idea, but do not demonstrate, that MMP1 is a substrate. Some TEPs inhibit targets through forming covalent bonds, while other TEPs use a noncovalent mechanism for protease inhibition, which hampers biochemical identification of substrates (*35*, *60*). A related unanswered question is the identity of the DAMP processed by MMP1 to induce macrophage attachment. One possibility, suggested by the requirement for Col4 and Laminin in this MMP1-dependent phenotype, is that fly macrophages detect digested BM fragments. Mammalian macrophages can sense such fragments as DAMPs through receptors such as RAGE (receptor for advanced glycation end-products) and CD44 that have no obvious homolog in flies, as well as integrins, CD36, and TLR2/4 (Toll-like receptor 2/4), which do have fly homologs (*44*, *45*, *61*). The identification of Tep3 and MMP1 substrates in this inflammatory axis is an important future goal.

This work highlights a number of similarities between Tep3 and its human ortholog, CD109, which is also the sole GPI-linked TEP in its species [reviewed in (*43*, *62*); orthology called via DIOPT (*63*)]. CD109 is up-regulated in tissue damage and is overexpressed in various carcinomas as well as glioblastoma; high CD109 levels indicate poor prognosis in multiple cancers. Experimental overexpression of CD109 decreases inflammation, while CD109 mutant mice show excess immune cell infiltration. CD109 phenotypes also point to an extracellular matrix–regulating role: Mutant mice exhibit fibrosis, while overexpression increases collagen organization. These effects have been largely attributed to inhibition of TGFB (transforming growth factor–β) signaling through direct interaction with both receptors and ligands, but this is not the case in all cell types. The fly results suggest that MMP antagonism by CD109 also merits examination.

Our data extend the cellular immune response to tumors to genetically engineered adult flies and demonstrate that in the OC model, macrophages have antitumor activity. Ablation of macrophages in these animals accelerates progression, while a condition that recruits more macrophages to the tumor slows progression. Previous work in larval and allograft models has revealed varying roles for macrophages, from protumor to antitumor to not being involved (*11*, *13*, *14*–*25*). Suggested mechanisms of antitumor macrophage activity include death caused by the tumor necrosis factor Eiger, ROS, or efferocytosis of transformed cells. However, we do not see enhanced cell death in high-grade OC tumors depleted for Tep3 (fig. S6, A to C), and death is not restricted to TAM-proximal sites. OC progression involves in part increased tumor invasiveness, and one role of macrophages in wound repair is to secrete BM components. Thus, a repair-like contribution of BM components is one potential mechanism by which TAMs could limit tumor progression.

Similarities as well as differences between human and fly tumors are revealed by this study. In both organisms, infiltration by immune cells is a positive indicator of an effective antitumor response, and such infiltration can negatively correlate with extracellular matrix levels in the tumor microenvironment. However, in humans, macrophages are often present in the tumor microenvironment of “cold” tumors that exclude tumorlytic T cells (*3*, *4*). In the fly, where macrophages are the sole immune cells, OC TAM numbers correlate with the strength of the antitumor response and host prognosis. In addition, the best-known DAMPs produced by human wounds and tumors are cytosolic molecules released by necrotic death, stimulating an inflammatory reaction, whereas apoptosis is generally considered immunosuppressive (*44*). Blocking apoptosis did not influence the TAM presence in the OC model, and depleting Tep3 in OC tumors did not increase necrosis (fig. S6, D to F); there is little information about cytosolic DAMPs in flies. Notably, the DAMP implicated by our work—BM damage through JNK-regulated MMP production—is agnostic to the type of cell death caused by a lesion but is one of many common features shared by both tumors and wounds (*52*, *53*). In wounds, JNK signaling resolves as tissue repair is completed, but in fly tumors, JNK is constitutively driven by oncogenic activity. In the OC model, this leads to constant high levels of Tep3, which blunts the antitumor immune response. Thus, tumors can exploit a physiological axis that reduces inflammatory cell association to promote their progression and kill the host more rapidly.

## MATERIALS AND METHODS

### Fly husbandry and genotypes

Most flies were maintained at 21°C in wide vials on cornmeal, molasses, and yeast food. OC flies were treated as described (*26*). For MMP1 or Rpr overexpression in WT ovaries, adult females were collected upon eclosion, maintained at 21°C for 2 days, then moved to new food, and shifted to 29°C for 1 day. *Tep3* RNAi construct no. 63660 from BDSC was used, except in fig. S2C. In RNAi or overexpression experiments, controls were used with matched *attP* sites targeting green fluorescent protein (GFP) or another exogenous protein. Complete genotypes are provided in table S1. For *Tep3* mutant alleles, *Tep3 sk2* is a 23–bp (base pair) deletion that creates a frameshift in the fifth exon, terminating after the first quarter of the protein; *Tep3-GAL4* is a CRIMIC (CRISPR-mediated integration cassette) inserted in the first intron, which is expected to terminate the protein shortly after initiation; *Tepq*^Δ^ deletes ∼1.5 kb of intragenic sequence ∼250 bp 5′ to the *Tep3* start codon (*40*).

For the LexA-driven OC model, the sequence encoding the chimeric transcriptional activator LexA-Hinge-GAL4 Activation Domain (LHG) was inserted using CRISPR-Cas9 homology-directed repair at the *tj* locus in the same site as the widely used *tj-GAL4* insertion. This chromosome was then recombined with *tubGAL80ts. LexAop-aPKCact+RasV12* was created by cloning sequences encoding aPKCdelN and Ras85DV12, separated by the T2A self-cleaving peptide, into *pJFRC19*. The transgene was inserted into the *attP2* site. To build *Hml^P2A^-LHG*, the *Hml^P2A^* promoter sequence from the *pHML-P2A-Gal4* plasmid [gift from A. Herzig (*28*)] was polymerase chain reaction amplified and cloned into *pattB-LHG* (gift from L. Mathies). The transgene was inserted into the *su(Hw)attP8* site on the X chromosome. To build *LexAop-NLSmScarlet3*, codon-optimized *3xNLS-mScarlet3* cDNA was synthesized by Integrated DNA Technologies and cloned into *pJFRC19*. The transgene was inserted into the *su(Hw)attP5* site on chromosome II and the *VK27* site on chromosome III. *UAS-Tep3-FLAG* places 3× FLAG tags at amino acid 35 into the *Tep3* coding sequence (*Drosophila* Genomics Resource Center) in the *pUASTattB* vector and was inserted into the *attP2* site via microinjection (BestGene Inc).

### Ovarian tumor induction, life-span assays, and tumor progression

Upon eclosion, adult females were placed at 21°C on food with yeast powder for 2 days. A maximum of 25 flies were kept in each vial. Flies were then moved to new food and shifted to 29°C to initiate tumor induction. Flies were transferred to new food every 2 days, and the number of dead flies was counted. For each life-span assay, at least 50 flies were used for each sample group, with control and experimental groups assessed contemporaneously. OC tumors were graded as described previously (*26*) on a scale of 1 to 4 with increasing severity.

### Immunofluorescence

Tissues were dissected in phosphate-buffered saline (PBS) and fixed in 4% PFA (paraformaldehyde)-PBS for 1 hour at room temperature without agitation. Samples were washed three times with PBS-TX (0.1% Triton-X in 1× PBS). For primary antibody staining, samples were blocked in 2% bovine serum albumin for 30 to 60 min. Samples were incubated overnight in a primary antibody at 4°C at the following concentrations: NimC1 (1:100), cDcp1 (1:100), MMP1 (1:100), Laminin (1:500), and Flag M2 (1:100). Samples were washed three times with PBS-TX and incubated with Alexa Fluor–conjugated secondary antibodies for 90 min at room temperature. To stain actin, fixed tissues were incubated for 60 to 90 min at room temperature with rhodamine-phalloidin at a concentration of 1:500. To stain nuclei, a 1:5000 4′,6-diamidino-2-phenylindole (DAPI) solution was applied for 15 min. After all incubations, samples were washed three times with PBS. Samples were mounted in Diamond Antifade Mountant before imaging. For ROS visualization, dihydroethidium (DHE) staining was performed as described previously (*46*). Dissected unfixed tissues were incubated in a 30 μM DHE solution for 5 min and washed three times with PBS, followed by mounting and imaging immediately. To visualize necrosis, dissected unfixed tissues were incubated in 15 μM propidium iodide for 10 min, washed three times with PBS, and immediately mounted and imaged.

### Protease activity assays

Tissues were rapidly dissected in Schneider’s media and transferred into a solution of casein-FITC (100 μg/ml) in media. Samples in tubes were gently agitated for 25 min, washed with PBS, and fixed in 4% PFA. Following further washing, samples were stained with a primary antibody (if necessary) or only phalloidin and DAPI. Samples were imaged within 72 hours to avoid degradation. The casein-FITC signal on tissues was measured using FIJI, and mean intensity values were quantified.

### Disc wounds

For ex vivo wounding, wing discs were dissected from third-instar larvae in culture medium [Schneider’s medium supplemented with 15% fetal bovine serum and penicillin/streptomycin (10,000 U/ml)], physically pinched by sharp forceps, and incubated at 29°C in the same culture medium for 3 hours before fixation, protease activity assays, or PI staining. For in vivo wounding, third-instar larval wing discs expressing *en-Gal4>UAS-RFP* or *nub-Gal4>UAS-GFP* were bisected by pressing the larval cuticle with a tungsten needle in cold PBS under a GFP/RFP (red fluorescent protein) fluorescence microscope (*54*). After wounding, larvae were transferred to food vials and maintained at 29°C for 6 hours before dissection.

### Embryo collection and larval counting

Embryos laid in 24 hours at 25°C were collected on agar + grape juice plates with yeast paste. Plates were aged at 25°C for 24 hours to allow larvae to develop. L1 larvae were counted with a GFP fluorescence microscope to score for the presence of the GFP balancer.

### Macrophage signal measurement

The NimC1 signal on ovaries (excluding vitellogenic follicles) or wing discs was measured using FIJI. A threshold was set using FIJI’s Threshold function to remove the background signal, and the resulting area of the NimC1 signal was quantified. The relative area was then calculated by dividing each value by the mean value of the control group. Signals calculated in this way correlate well with counts of individual macrophage nuclei (fig. S6G). Individual macrophage nuclei were counted using FIJI’s Threshold function to remove the background signal. FIJI’s Watershed function was then used to segment nuclei, and particles of the appropriate size (12 to 60 μm^2^) with a circularity of 0.3 to 1.0 were counted using the Analyze Particles function.

### Apoptosis and necrosis measurement

cDcp1 (apoptosis) and PI (necrosis) signals in nonvitellogenic follicles were quantified using FIJI. For cDcp1, a threshold was set using FIJI’s Threshold function to remove the background signal, and the resulting cDcp1-positive area was measured. The relative area was calculated by dividing each value by the mean value of the control group. For PI, the mean intensity was measured.

### BM, MMP1, and ROS measurement

The mean intensity of Vkg-GFP (Col4GFP), LanB-GFP, DHE, and antibody signals against Laminin and MMP1 was measured using FIJI. The normalized signal was calculated by dividing each value by the mean value of the control group.

### Confocal microscopy and image analysis

Fixed samples were imaged on the Zeiss LSM900 Confocal Microscope with a Plan-APOCHROMAT 20×/0.8 objective. Images were processed and analyzed with FIJI software. Unless otherwise indicated, all images are maximum projections. Maximum projection images were created in FIJI software. Figures were assembled in Adobe Illustrator using multiple panels, where merited.

### Statistics

A one-way analysis of variance (ANOVA) test, Student’s unpaired *t* test, or Student’s paired *t* test was used for parametric data. A Mann-Whitney *U* test and Kruskal-Wallis test were used for tumor progression. A log-rank test was used to determine significant differences in survival curves. An exact binomial test was used to test for deviation from the expected Mendelian ratio of 1:1. GraphPad Prism was used to perform statistical testing. All error bars show SEM, except in Fig. 5D, where they show the 95% confidence interval.
