# Supplementary material for: Macrophage restriction in Drosophila wounds and tumors via a matrix degradation–moderating protease inhibitor
Source: Sci Adv. 2026 Sep 25;12(39):eaec5131. doi: 10.1126/sciadv.aec5131 (PMC13614384; doi:10.1126/sciadv.aec5131)
Supplement: Supplementary file 1 [file sciadv.aec5131_sm.pdf]

Science Advances
AAAS

# Supplementary Materials for

## Macrophage restriction in *Drosophila* wounds and tumors via a matrix degradation–moderating protease inhibitor

Yoshiki Sakai *et al.*

Corresponding author: David Bilder, bilder@berkeley.edu

*Sci. Adv.* **12**, eaec5131 (2026)
DOI: 10.1126/sciadv.aec5131

**This PDF file includes:**

Figs. S1 to S6
Tables S1 and S2

**Fig. S1.**

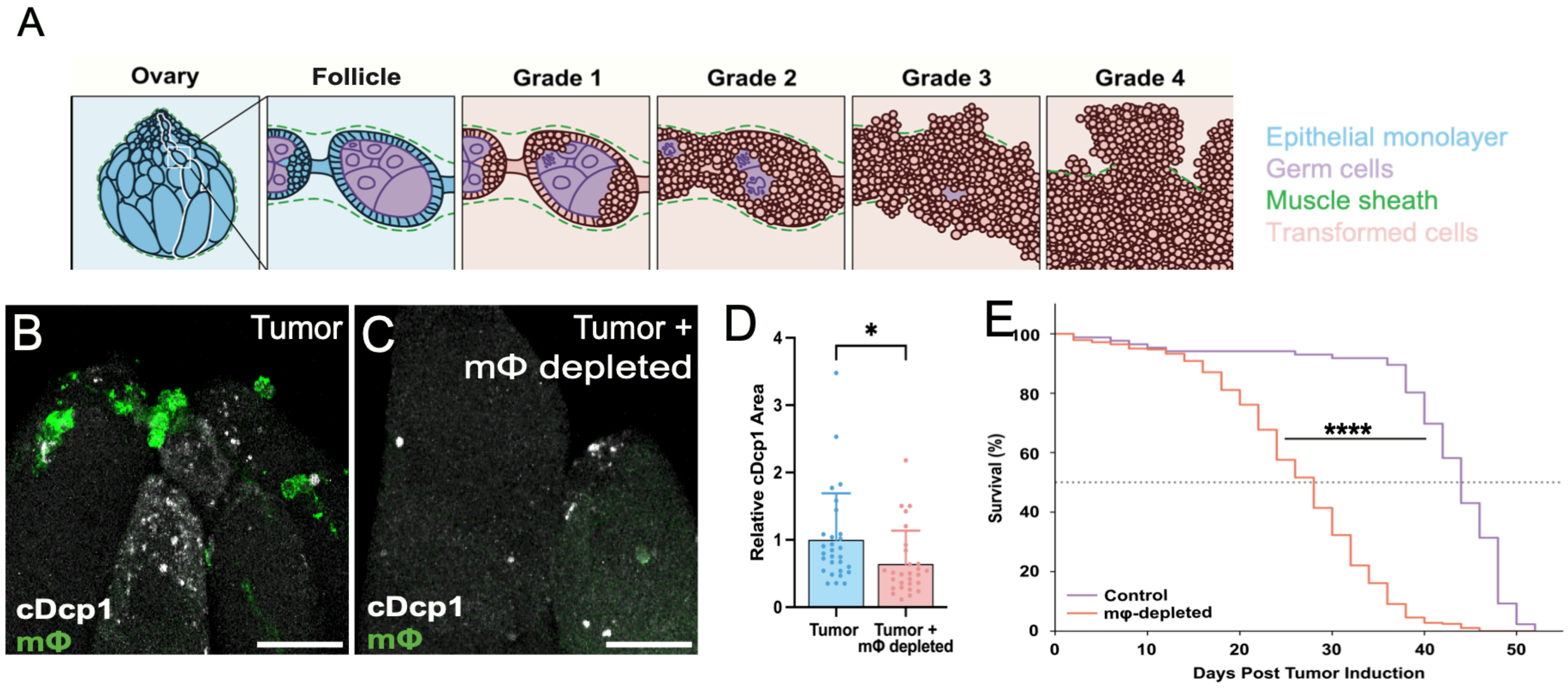


**Figure S1: (A)** Schematic of tumor progression in ovarian carcinoma (OC) tumor model, modified from Hsi & Ong et al. (*26*). Grade 1 tumors exhibit loss of epithelial polarity and overproliferation but do not disrupt follicle organization. Grade 2 tumors display fusion between follicles and absence of germ cells. Grade 3 tumors break down the muscle sheath and cause fusion between neighboring ovarioles. Grade 4 tumors display further disruption of ovariole morphology and organ-wide basement membrane breakdown. **(B, C)** Tumors in macrophage-depleted hosts show slightly reduced apoptosis, assayed by cDcp1 staining (white), quantified in **(D)** (n-values: Tumor = 29, Tumor + mφ-depleted = 28). **(E)** Depleting macrophages in non-tumor-bearing hosts decreases lifespan (n-values: Control = 86, mφ-depleted = 285). Scale bars = 50µm; Unpaired t-test (D), Log-rank test (E) used to determine significance. *p < .05, ***p < .0005. Dots in (D) represent individual samples.

**Fig S2.**

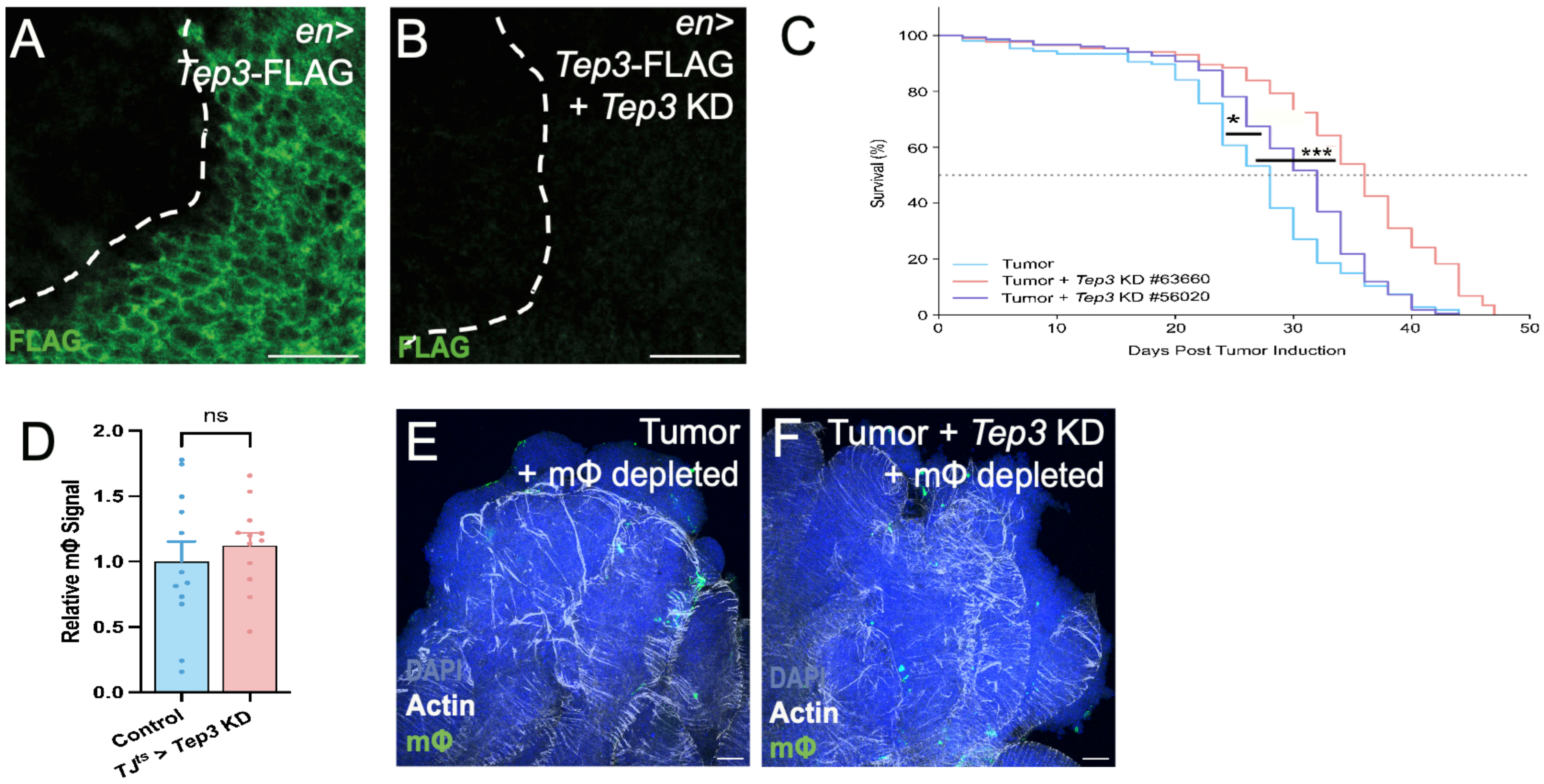


**Figure S2: (A, B)** Tep3 is localized to the plasma membrane, and *Tep3* RNAi effectively depletes transgenic Tep3-FLAG driven by *enGAL4* in imaginal discs (green, single confocal slice, *en*-positive region marked by dotted white line). **(C)** Tumor-specific knockdown of *Tep3* with either of two RNAi constructs extends host lifespan (n-values: Tumor = 107, Tumor + *Tep3* KD #63660 = 101, Tumor + *Tep3* KD #56020 = 151). **(D)** *Tep3* KD in WT ovaries does not affect macrophage association (n-values: Control =12, $TJ^{ts}$>*Tep3* KD = 12). **(E, F)** *Tep3* KD tumors in macrophage-depleted hosts no longer exhibit slowed tumor progression (n-values: mφ-depleted Tumor = 39, mφ-depleted Tumor + *Tep3* KD = 71). Scale bars = 20µm (A, B), 50 µm (E, F); Log-rank test (C) and unpaired t-test (D) used to determine significance; *p < .05, ***p < .0005. Dots in (D) represent individual samples.

**Fig. S3.**

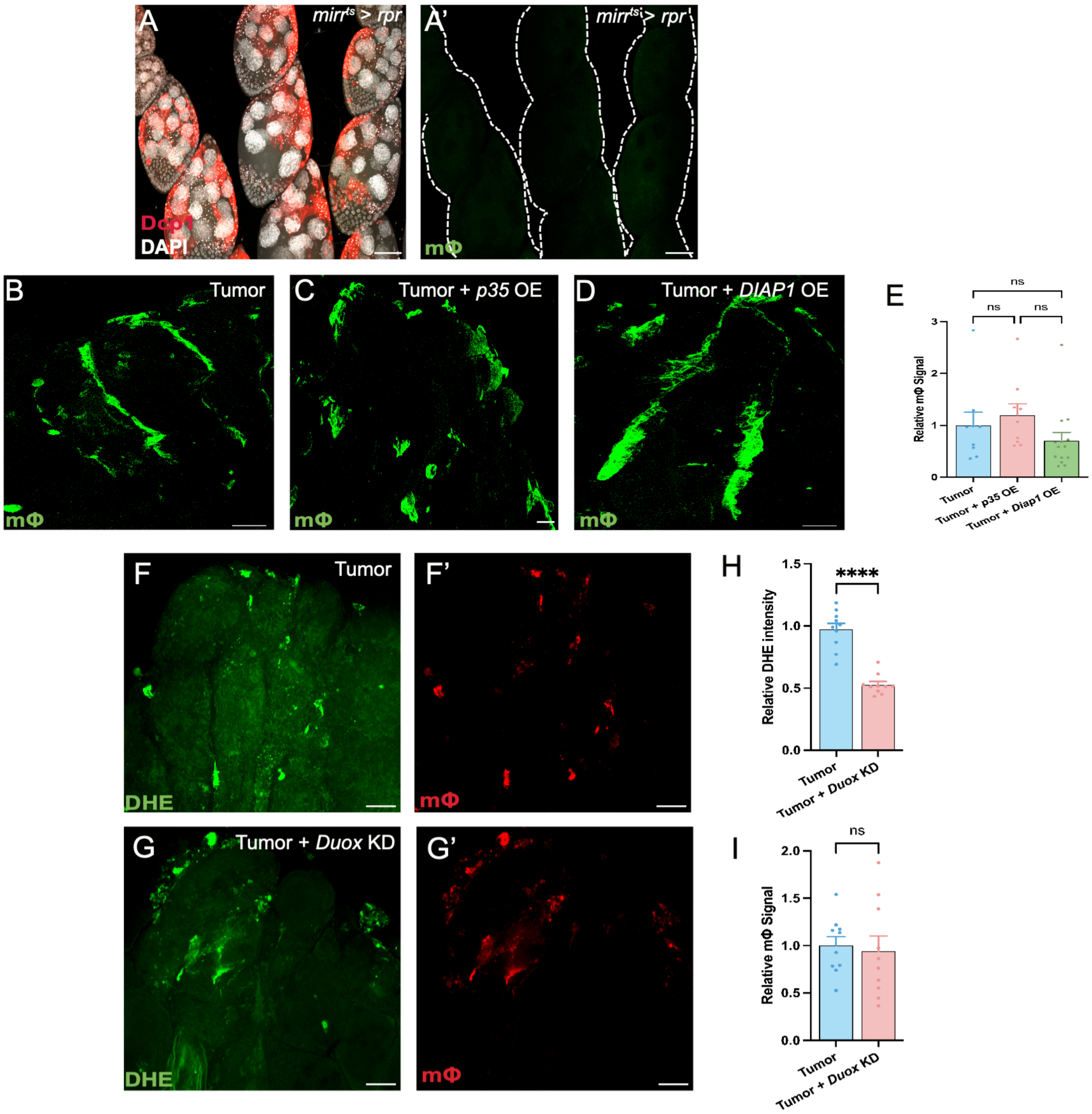


**Figure S3: (A)** Expression of the apoptotic regulator Rpr in follicle cells induces apoptosis but is not sufficient to recruit macrophages. **(B-D)** Inhibiting apoptosis in tumors does not affect macrophage recruitment, quantitated in **(E)** (n values: Tumor = 9, Tumor + *p35* OE = 9, Tumor + *Diap1* OE = 14). **(F-G)** Expressing *Duox* RNAi in tumors decreases ROS levels but does not change macrophage association, assayed by *Pxn-LexA>LexAop-mCherry*, quantitated in **H, I** (n values: Tumor = 10, Tumor + *Duox* KD = 10). Scale bars = 50μm; Unpaired t-test (H, I) and One Way ANOVA (E) used to determine significance; ****p < .00005. Dots in (E, H, I) represent individual samples.

**Fig. S4.**

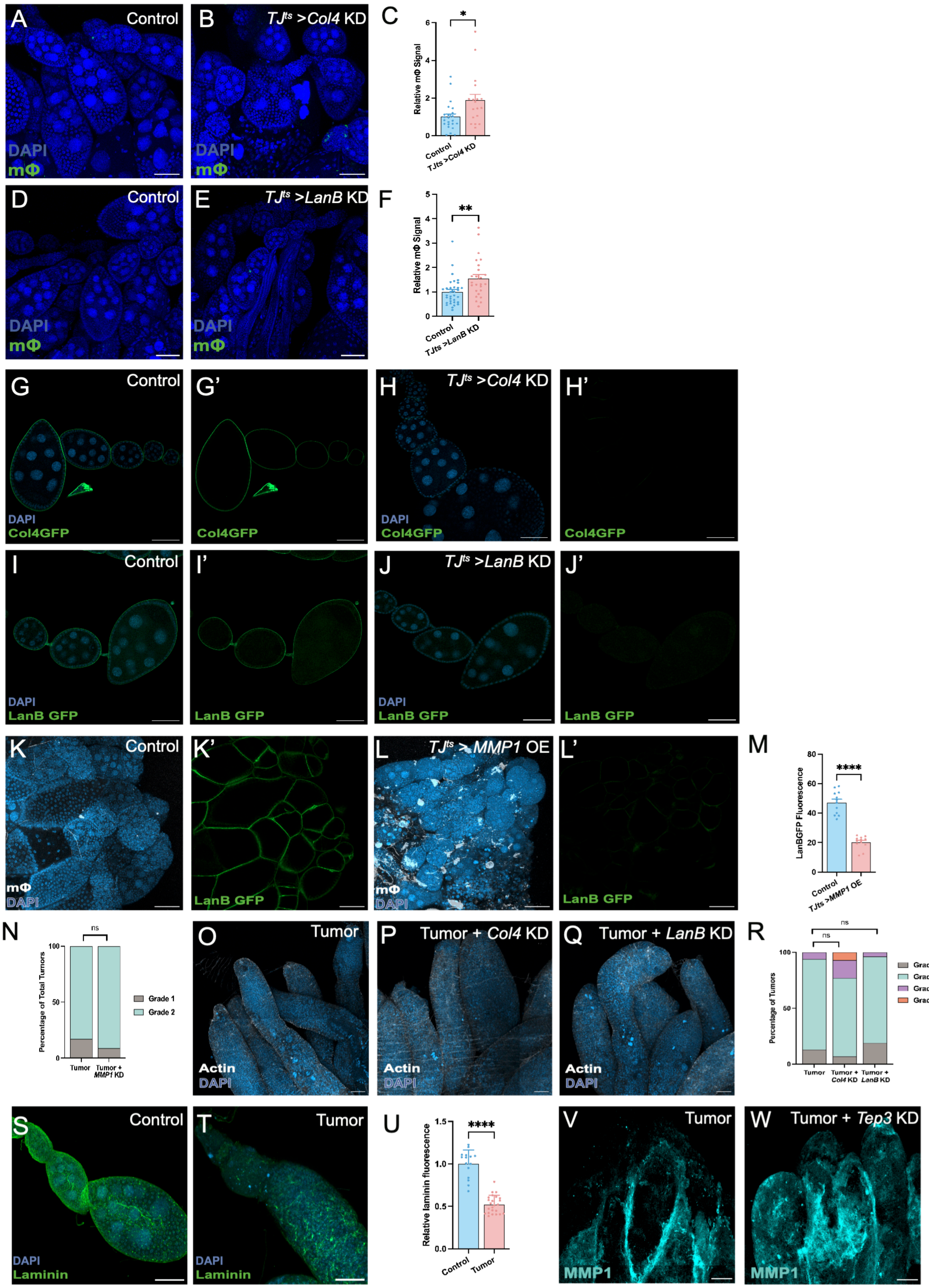
A Control
B TJts >Col4 KD
C
D Control
E TJts >LanB KD
F
DAPI
mΦ
Relative mΦ Signal
Control
TJts >Col4 KD
TJts >LanB KD
G Control
G'
H TJts >Col4 KD
H'
Col4GFP
I Control
I'
J TJts >LanB KD
J'
LanB GFP
K Control
K'
L TJts > MMP1 OE
L'
M
LanBGFP Fluorescence
TJts >MMP1 OE
N
Percentage of Total Tumors
Grade 1
Grade 2
Tumor
Tumor + MMP1 KD
O Tumor
P Tumor + Col4 KD
Q Tumor + LanB KD
Actin
R
Percentage of Tumors
Grade 3
Grade 4
Tumor + Col4 KD
Tumor + LanB KD
ns
S Control
T Tumor
Laminin
U
Relative laminin fluorescence
V Tumor
W Tumor + Tep3 KD
MMP1

**Figure S4:** WT ovaries with *Col4* KD **(A, B)** or *LanB* KD **(D, E)** show slight increase in macrophage recruitment (green), quantitated in **C** (n values: Ovary = 23, Ovary + *Col4* KD = 18) and **F** (n values: Ovary = 33, Ovary + *LanB* KD = 25). Efficiency of each RNAi is validated by loss of vkg-GFP (Col4GFP) **(G, H)** and LanB-GFP **(I, J)**. **(K, L)** MMP1 overexpression in WT ovaries causes laminin degradation, as shown by reduced LanB-GFP, quantified in **(M)** (n-values: Control = 12, *TJ*$^{ts}$*>MMP1 OE* = 12), and promotes macrophage recruitment. **(N)** MMP1 depletion in tumors does not affect tumor progression at day 10 ATI (n values: Tumor = 23, Tumor + *MMP1* KD = 33). **(O-Q)** Depletion of Col4 or laminin has no effect on tumor progression at day 10 ATI, quantified in **(R)** (n-values: Tumor = 32, Tumor + *Col4* KD = 30, Tumor + *LanB* KD = 26). **(S, T)** Laminin levels are reduced in tumors compared to WT ovaries, quantified in **(U)** (n-values: Control = 15, Tumor = 24). **(V, W)** Tep3 depletion in tumors causes a slight increase in MMP1 protein levels, assayed by anti-MMP1 antibody, quantified in Fig. 4H. Scale bars = 50µm; Unpaired t-test (C, F, M, U), Mann-Whitney U-test (N), Kruskal-Wallis test (R) used to determine significance. *p < .05, **p < .005, ****p < .00005. Dots in (C, F, M, U) represent individual samples.

**Fig. S5.**

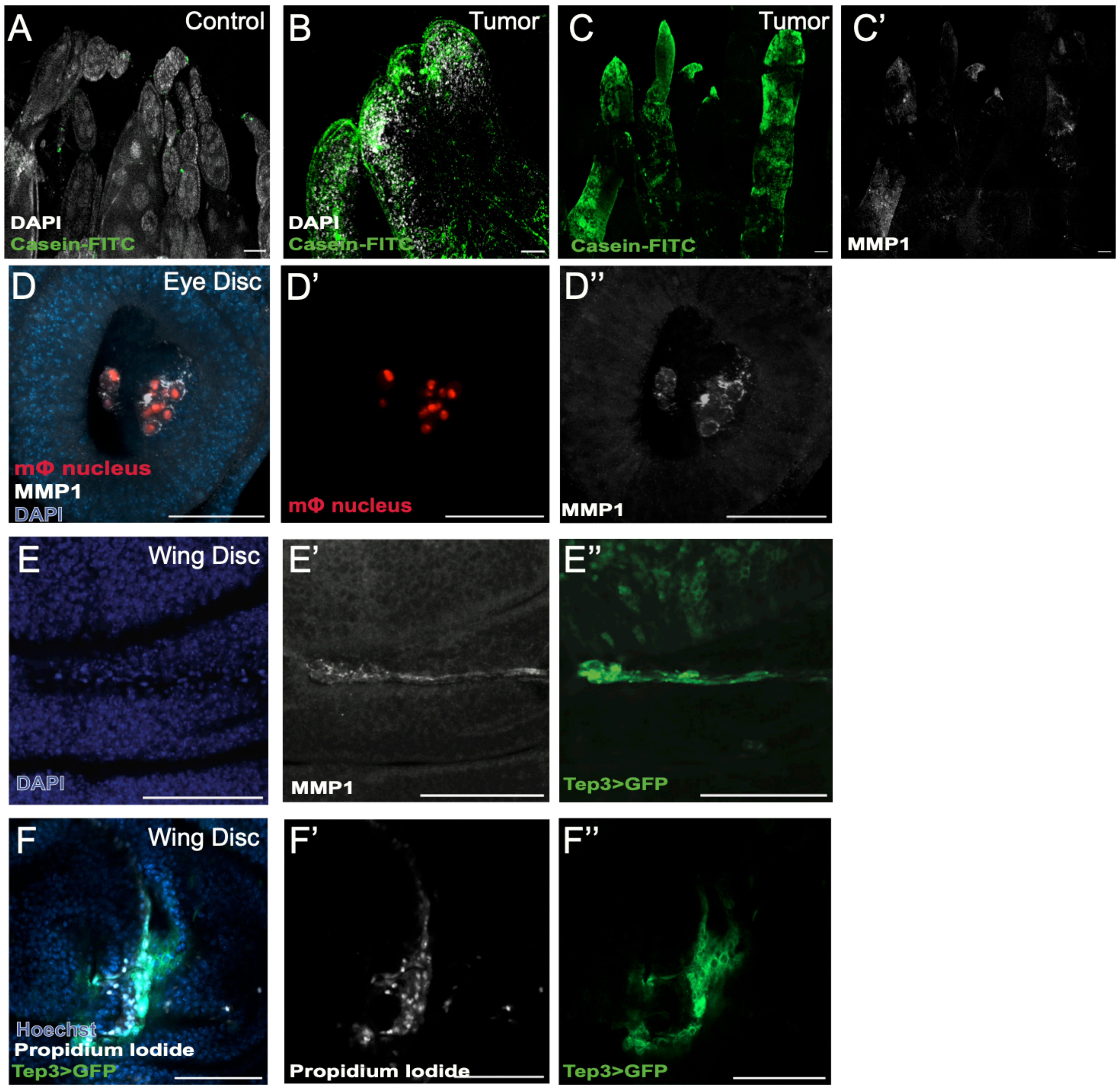


**Figure S5: (A, B)** Tumors display increased Casein-FITC fluorescence (green) compared to WT ovaries. Tumor Casein-FITC signal (green) **(C)** is correlated with MMP1 expression (white). **(D)** Macrophages (marked by *Hml$^{P2A}$-LHG>LexAop-NLSmScarlet3* nuclear marker) on eye disc naturally express MMP1 (white). **(E)** Closeup of injured wing disc from Fig. 6B. Wound expresses MMP1 (white), detected by antibody, and *Tep3* (green), detected by transcriptional reporter. **(F)** *Tep3* induction (green) upon wounding colocalizes with necrotic cells labeled with Propidium Iodide (white). Scale bars = 50µm.

**Fig. S6.**

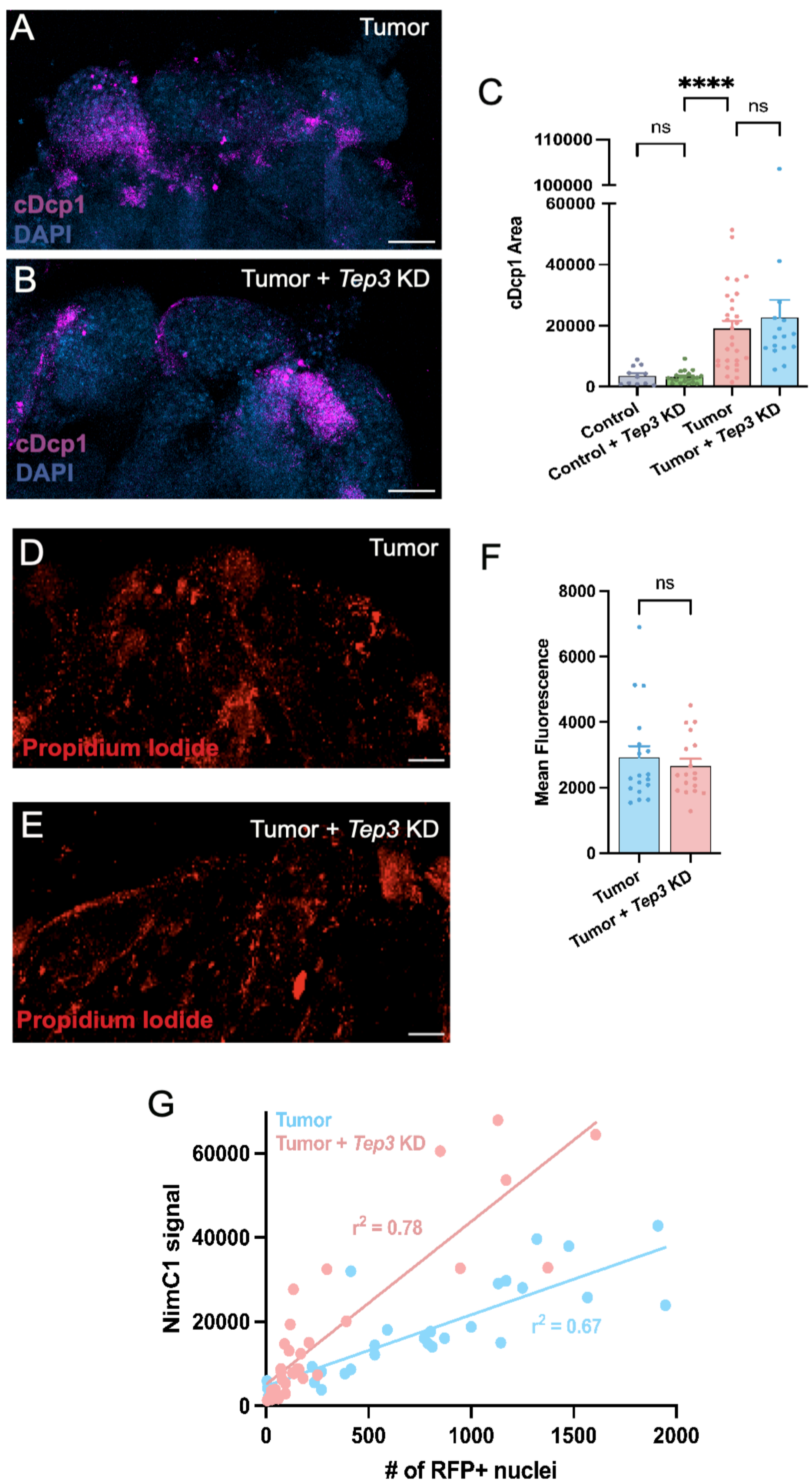


**Figure S6: (A, B)** At day 20 ATI, *Tep3* KD does not change the amount of apoptosis in the tumor, indicated by cDcp1 staining (pink), quantitated in **(C)** (n values: Control = 11, Control + *Tep3* KD = 21, Tumor = 29, Tumor + *Tep3* KD = 16). **(D, E)** *Tep3* KD in tumors does not alter necrosis, detected by Propidium Iodide (red), quantitated in **F** (Tumor = 19, Tumor + *Tep3* KD = 18). **(G)** Correlation plot comparing NimC1 fluorescence area to number of mScarlet-positive macrophage nuclei (*Hml$^{P2A}$-LHG>LexAop-NLSmScarlet3*) in the same sample. Colors indicate sample genotypes (n values: Tumor = 28, Tumor + *Tep3* KD = 33). Scale bars = 50µm; Unpaired t-test (C, F), linear progression analysis (G) used to determine significance. Dots in (C, F) represent individual samples

# Table S1.

| Figure | Panel | Condition | Genotype |
|---|---|---|---|
| 1 | B-D | Control | Hml$^{P2A}$-LHG/+; tj-Gal4, tubulin-Gal80$^{ts}$/LexAop-3xnls::mScarlet3; +/+ |
| | | Tumor | Hml$^{P2A}$-LHG/+; tj-Gal4, tubulin-Gal80$^{ts}$/LexAop-3xnls::mScarlet3; UAS-aPKC$^{\Delta N}$, UAS-Ras$^{V12}$/+ |
| | E-I | Tumor | tj-Gal4, tubulin-Gal80$^{ts}$/+; UAS-aPKC$^{\Delta N}$, UAS-Ras$^{V12}$/+ |
| | J-M | Tumor | tj-LHG, tubulin-Gal80$^{ts}$/+; LexAop-aPKC$^{\Delta N}$-T2A-Ras$^{V12}$, Hml$^{P2A}$-Gal4/+ |
| | | Tumor + mφ depleted | tj-LHG, tubulin-Gal80$^{ts}$/UAS-hid:UAS-rpr; LexAop-aPKC$^{\Delta N}$-T2A-Ras$^{V12}$, Hml$^{P2A}$-Gal4/+ |
| 2 | B-D | Control | tubulin-Gal80$^{ts}$/Tep3-Gal4; +/UAS-mCD8GFP |
| | | Tumor | tj-LHG, tubulin-Gal80$^{ts}$/Tep3-Gal4; LexAop-aPKC$^{\Delta N}$-T2A-Ras$^{V12}$/UAS-mCD8GFP |
| | E-G, I-K | Control | tj-Gal4, tubulin-Gal80$^{ts}$/UAS-GFP KD; +/+ |
| | | Tumor | tj-Gal4, tubulin-Gal80$^{ts}$/UAS-GFP KD; UAS-aPKC$^{\Delta N}$, UAS-Ras$^{V12}$/+ |
| | | Tumor + Tep3 KD | tj-Gal4, tubulin-Gal80$^{ts}$/UAS-Tep3 KD; UAS-aPKC$^{\Delta N}$, UAS-Ras$^{V12}$/+ |
| | H | Tumor | tj-Gal4, tubulin-Gal80$^{ts}$/UAS-GFP KD; UAS-aPKC$^{\Delta N}$, UAS-Ras$^{V12}$/LexAop-rpr |
| | | Tumor + Tep3 KD | tj-Gal4, tubulin-Gal80$^{ts}$/UAS-Tep3 KD; UAS-aPKC$^{\Delta N}$, UAS-Ras$^{V12}$/LexAop-rpr |
| | | Tumor + mφ depleted | Hml$^{P2A}$-LHG/+; tj-Gal4, tubulin-Gal80$^{ts}$/UAS-GFP KD; UAS-aPKC$^{\Delta N}$, UAS-Ras$^{V12}$/LexAop-rpr |
| | | Tumor + Tep3 KD + mφ depleted | Hml$^{P2A}$-LHG/+; tj-Gal4, tubulin-Gal80$^{ts}$/UAS-Tep3 KD; UAS-aPKC$^{\Delta N}$, UAS-Ras$^{V12}$/LexAop-rpr |
| 3 | A-C | Tumor | tj-Gal4, tubulin-Gal80$^{ts}$/UAS-GFP KD; UAS-aPKC$^{\Delta N}$, UAS-Ras$^{V12}$/+ |
| | | Tumor + Tep3 KD | tj-Gal4, tubulin-Gal80$^{ts}$/UAS-Tep3 KD; UAS-aPKC$^{\Delta N}$, UAS-Ras$^{V12}$/+ |
| | D-F | Control | tj-Gal4, tubulin-Gal80$^{ts}$/vkg-GFP; +/+ |
| | | TJts>MMP1 OE | tj-Gal4, tubulin-Gal80$^{ts}$/vkg-GFP; UAS-MMP1/+ |
| | H-J | Tumor | tj-Gal4, tubulin-Gal80$^{ts}$/+; UAS-aPKC$^{\Delta N}$, UAS-Ras$^{V12}$/UAS-mCherry KD |
| | | Tumor + MMP1 KD | tj-Gal4, tubulin-Gal80$^{ts}$/+; UAS-aPKC$^{\Delta N}$, UAS-Ras$^{V12}$/UAS-MMP1 KD |
| | K-M | Tumor | tj-Gal4, tubulin-Gal80$^{ts}$/UAS-GFP KD; UAS-aPKC$^{\Delta N}$, UAS-Ras$^{V12}$/+ |
| | | Tumor + Col4 KD | tj-Gal4, tubulin-Gal80$^{ts}$/UAS-vkg KD; UAS-aPKC$^{\Delta N}$, UAS-Ras$^{V12}$/UAS-cg25c KD |
| | N-P | Tumor | tj-Gal4, tubulin-Gal80$^{ts}$/UAS-GFP KD; UAS-aPKC$^{\Delta N}$, UAS-Ras$^{V12}$/+ |
| | | Tumor + lanB KD | tj-Gal4, tubulin-Gal80$^{ts}$/UAS-lanB2 KD; UAS-aPKC$^{\Delta N}$, UAS-Ras$^{V12}$/UAS-lanB1 KD |
| 4 | A-D | Tumor | tj-Gal4, tubulin-Gal80$^{ts}$/UAS-GFP KD; UAS-aPKC$^{\Delta N}$, UAS-Ras$^{V12}$/+ |
| | | Tumor + MMP1 KD | tj-Gal4, tubulin-Gal80$^{ts}$/UAS-GFP KD; UAS-aPKC$^{\Delta N}$, UAS-Ras$^{V12}$/UAS-MMP1 KD |
| | | Tumor + Tep3 KD | tj-Gal4, tubulin-Gal80$^{ts}$/UAS-Tep3 KD; UAS-aPKC$^{\Delta N}$, UAS-Ras$^{V12}$/+ |
| | E-H | Tumor | tj-Gal4, tubulin-Gal80$^{ts}$/UAS-GFP KD; UAS-aPKC$^{\Delta N}$, UAS-Ras$^{V12}$/+ |
| | | Tumor + Tep3 KD | tj-Gal4, tubulin-Gal80$^{ts}$/UAS-Tep3 KD; UAS-aPKC$^{\Delta N}$, UAS-Ras$^{V12}$/+ |
| | I-L, S, T | Tumor | tj-Gal4, tubulin-Gal80$^{ts}$/UAS-GFP KD; UAS-aPKC$^{\Delta N}$, UAS-Ras$^{V12}$/+ |
| | | Tumor + Tep3 KD | tj-Gal4, tubulin-Gal80$^{ts}$/UAS-Tep3 KD; UAS-aPKC$^{\Delta N}$, UAS-Ras$^{V12}$/+ |
| | | Tumor + MMP1 KD | tj-Gal4, tubulin-Gal80$^{ts}$/UAS-GFP KD; UAS-aPKC$^{\Delta N}$, UAS-Ras$^{V12}$/UAS-MMP1 KD |
| | | Tumor + MMP1 KD + Tep3 KD | tj-Gal4, tubulin-Gal80$^{ts}$/UAS-Tep3 KD; UAS-aPKC$^{\Delta N}$, UAS-Ras$^{V12}$/UAS-MMP1 KD |
| | M, N, U | Control | tubulin-Gal80$^{ts}$/+; Hml$^{P2A}$-Gal4/UAS-mCherry KD |
| | | Hml$^{P2A}$>Tep3 OE | tubulin-Gal80$^{ts}$/+; Hml$^{P2A}$-Gal4/UAS-Tep3-FLAG |
| | O-R, V | Tumor | tj-Gal4, tubulin-Gal80$^{ts}$/UAS-GFP KD; UAS-aPKC$^{\Delta N}$, UAS-Ras$^{V12}$/UAS-luciferase |
| | | Tumor + Tep3 KD | tj-Gal4, tubulin-Gal80$^{ts}$/UAS-Tep3 KD; UAS-aPKC$^{\Delta N}$, UAS-Ras$^{V12}$/UAS-luciferase |
| | | Tumor + Timp OE | tj-Gal4, tubulin-Gal80$^{ts}$/UAS-GFP KD; UAS-aPKC$^{\Delta N}$, UAS-Ras$^{V12}$/UAS-Timp |
| | | Tumor + Tep3 KD + Timp OE | tj-Gal4, tubulin-Gal80$^{ts}$/UAS-Tep3 KD; UAS-aPKC$^{\Delta N}$, UAS-Ras$^{V12}$/UAS-Timp |
| 5 | A | Trachea | w$^{1118}$ |
| | B | Imaginal Ring | w$^{1118}$ |
| | D | Tepq$^{\Delta}$/CyO-Tb | Tepq$^{\Delta}$/CyO-Tb; +/+ (F1 progeny from Tepq$^{\Delta}$/Tepq$^{\Delta}$; +/+ x Tep3$^{sk2}$/CyO-Tb; +/+) |
| | | Tepq$^{\Delta}$/Tep3$^{sk2}$ | Tepq$^{\Delta}$/Tep3$^{sk2}$; +/+ (F1 progeny from Tepq$^{\Delta}$/Tepq$^{\Delta}$; +/+ x Tep3$^{sk2}$/CyO-Tb; +/+) |
| | F-H | Control | w$^{1118}$ |
| | | Tepq$^{\Delta}$/Tep3$^{sk2}$ | Tepq$^{\Delta}$/Tep3$^{sk2}$; +/+ |
| 6 | A, B | Unwounded/Wounded | Tep3-Gal4/+; UAS-mCD8GFP/+ |
| | C, D | Unwounded/Wounded | en-Gal4, UAS-RFP/+; Dr/+ |

| | | | |
|---|---|---|---|
| | E-G | Control | w$^{1118}$ |
| | | Tepq$^{\Delta}$/Tep3$^{sk2}$ | Tepq$^{\Delta}$/Tep3$^{sk2}$; +/+ |
| **7** | A-F | en>RFP + Tep3 OE | en-Gal4, UAS-RFP/+; Dr/UAS-Tep3-FLAG |
| | G-K | nub>GFP | nub-Gal4, UAS-GFP/+; +/+ |
| | | nub>GFP + Tep3 KD | nub-Gal4, UAS-GFP/UAS-Tep3 KD; +/+ |
| **S1** | B-D | Tumor | tj-LHG, tubulin-Gal80$^{ts}$/UAS-GFP KD; LexAop-aPKC$^{\Delta N}$-T2A-Ras$^{V12}$, Hml$^{P2A}$-Gal4/+ |
| | | Tumor + mφ depleted | tj-LHG, tubulin-Gal80$^{ts}$/UAS-hid:UAS-rpr; LexAop-aPKC$^{\Delta N}$-T2A-Ras$^{V12}$, Hml$^{P2A}$-Gal4/+ |
| | E | Control | tubulin-Gal80$^{ts}$/UAS-GFP KD; Hml$^{P2A}$-Gal4/+ |
| | | Control + mφ depleted | tubulin-Gal80$^{ts}$/UAS-hid:UAS-rpr; Hml$^{P2A}$-Gal4/+ |
| **S2** | A, B | en>Tep3-FLAG | en-Gal4, UAS-RFP/+; Dr/UAS-Tep3-FLAG |
| | | en>Tep3-FLAG + Tep3 KD | en-Gal4, UAS-RFP/UAS-Tep3 KD; Dr/UAS-Tep3-FLAG |
| | C | Tumor | tj-Gal4, tubulin-Gal80$^{ts}$/UAS-GFP KD; UAS-aPKC$^{\Delta N}$, UAS-Ras$^{V12}$/+ |
| | | Tumor + Tep3 KD #63660 | tj-Gal4, tubulin-Gal80$^{ts}$/UAS-Tep3 KD #63660; UAS-aPKC$^{\Delta N}$, UAS-Ras$^{V12}$/+ |
| | | Tumor + Tep3 KD #56020 | tj-Gal4, tubulin-Gal80$^{ts}$/UAS-Tep3 KD #56020; UAS-aPKC$^{\Delta N}$, UAS-Ras$^{V12}$/+ |
| | D | Control | tj-Gal4, tubulin-Gal80$^{ts}$/UAS-GFP KD; +/+ |
| | | TJ$^{ts}$>Tep3 KD | tj-Gal4, tubulin-Gal80$^{ts}$/UAS-Tep3 KD; +/+ |
| | E-F | Tumor + mφ depleted | Hml$^{P2A}$-LHG/+; tj-Gal4, tubulin-Gal80$^{ts}$/UAS-GFP KD; UAS-aPKC$^{\Delta N}$, UAS-Ras$^{V12}$/LexAop-rpr |
| | | Tumor + Tep3 KD + mφ depleted | Hml$^{P2A}$-LHG/+; tj-Gal4, tubulin-Gal80$^{ts}$/UAS-Tep3 KD; UAS-aPKC$^{\Delta N}$, UAS-Ras$^{V12}$/LexAop-rpr |
| **S3** | A | mirr$^{ts}$ >rpr | UAS-rpr, tubulin-Gal80$^{ts}$/+; mirr-Gal4/+ |
| | B-E | Tumor | tj-Gal4, tubulin-Gal80$^{ts}$/UAS-luciferase; UAS-aPKC$^{\Delta N}$, UAS-Ras$^{V12}$/+ |
| | | Tumor + p35 OE | tj-Gal4, tubulin-Gal80$^{ts}$/Sp; UAS-aPKC$^{\Delta N}$, UAS-Ras$^{V12}$/UAS-p35 |
| | | Tumor + DIAP1 OE | tj-Gal4, tubulin-Gal80$^{ts}$/+; UAS-aPKC$^{\Delta N}$, UAS-Ras$^{V12}$/UAS-DIAP1 |
| | F-I | Tumor | tj-Gal4, tubulin-Gal80$^{ts}$/Pxn-LexA, LexAop-mCherry; UAS-aPKC$^{\Delta N}$, UAS-Ras$^{V12}$/UAS-GFP KD |
| | | Tumor + Duox KD | tj-Gal4, tubulin-Gal80$^{ts}$/Pxn-LexA, LexAop-mCherry; UAS-aPKC$^{\Delta N}$, UAS-Ras$^{V12}$/UAS-Duox KD |
| **S4** | A-C | Control | tj-Gal4, tubulin-Gal80$^{ts}$/UAS-GFP KD; +/+ |
| | | TJ$^{ts}$>Col4 KD | tj-Gal4, tubulin-Gal80$^{ts}$/UAS-vkg KD; +/UAS-cg25c KD |
| | D-F | Control | tj-Gal4, tubulin-Gal80$^{ts}$/UAS-GFP KD; +/+ |
| | | TJ$^{ts}$>LanB KD | tj-Gal4, tubulin-Gal80$^{ts}$/UAS-lanB1 KD; +/UAS-lanB2 KD |
| | G, H | Control | tj-Gal4, vkg-GFP/+; tubulin-Gal80$^{ts}$/+ |
| | | TJ$^{ts}$>Col4 KD | tj-Gal4, vkg-GFP/UAS-vkg KD; tubulin-Gal80$^{ts}$/UAS-cg25c KD |
| | I, J | Control | tj-Gal4, tubulin-Gal80$^{ts}$/+; LanB1-GFP/+ |
| | | TJ$^{ts}$>LanB KD | tj-Gal4, tubulin-Gal80$^{ts}$/UAS-lanB2 KD; LanB1-GFP/UAS-lanB1 KD |
| | K-M | Control | tj-Gal4, tubulin-Gal80$^{ts}$/+; LanB1-GFP/+ |
| | | TJ$^{ts}$>MMP1 OE | tj-Gal4, tubulin-Gal80$^{ts}$/+; LanB1-GFP/UAS-MMP1 |
| | N | Tumor | tj-Gal4, tubulin-Gal80$^{ts}$/+; UAS-aPKC$^{\Delta N}$, UAS-Ras$^{V12}$/UAS-mCherry KD |
| | | Tumor + MMP1 KD | tj-Gal4, tubulin-Gal80$^{ts}$/+; UAS-aPKC$^{\Delta N}$, UAS-Ras$^{V12}$/UAS-MMP1 KD |
| | O-R | Tumor | tj-Gal4, tubulin-Gal80$^{ts}$/UAS-GFP KD; UAS-aPKC$^{\Delta N}$, UAS-Ras$^{V12}$/+ |
| | | Tumor + Col4 KD | tj-Gal4, tubulin-Gal80$^{ts}$/UAS-vkg KD; UAS-aPKC$^{\Delta N}$, UAS-Ras$^{V12}$/UAS-cg25c KD |
| | | Tumor + LanB KD | tj-Gal4, tubulin-Gal80$^{ts}$/UAS-lanB2 KD; UAS-aPKC$^{\Delta N}$, UAS-Ras$^{V12}$/UAS-lanB1 KD |
| | S-U | Control | tj-Gal4, tubulin-Gal80$^{ts}$/UAS-GFP KD; +/+ |
| | | Tumor | tj-Gal4, tubulin-Gal80$^{ts}$/UAS-GFP KD; UAS-aPKC$^{\Delta N}$, UAS-Ras$^{V12}$/+ |
| | V, W | Tumor | tj-Gal4, tubulin-Gal80$^{ts}$/UAS-GFP KD; UAS-aPKC$^{\Delta N}$, UAS-Ras$^{V12}$/+ |
| | | Tumor + Tep3 KD | tj-Gal4, tubulin-Gal80$^{ts}$/UAS-Tep3 KD; UAS-aPKC$^{\Delta N}$, UAS-Ras$^{V12}$/+ |
| **S5** | A-C | Control | tj-Gal4, tubulin-Gal80$^{ts}$/UAS-GFP KD; +/+ |
| | | Tumor | tj-Gal4, tubulin-Gal80$^{ts}$/UAS-GFP KD; UAS-aPKC$^{\Delta N}$, UAS-Ras$^{V12}$/+ |
| | D | Eye disc | Hml$^{P2A}$-LHG/+; LexAop-3xnls::mScarlet3/+; +/+ |
| | E | Wing disc | Tep3-Gal4/+; UAS-mCD8GFP/+ |
| | F | Wing disc | Tep3-Gal4/CyO; UAS-mCD8GFP/UAS-mCD8GFP |
| **S6** | A-C | Control | tj-Gal4, tubulin-Gal80$^{ts}$/UAS-GFP KD; +/+ |

| | | |
|---|---|---|
| | Control + Tep3 KD | *tj-Gal4, tubulin-Gal80$^{ts}$/UAS-Tep3 KD; +/+* |
| | Tumor | *tj-Gal4, tubulin-Gal80$^{ts}$/UAS-GFP KD; UAS-aPKC$^{\Delta N}$, UAS-Ras$^{V12}$/+* |
| | Tumor + Tep3 KD | *tj-Gal4, tubulin-Gal80$^{ts}$/UAS-Tep3 KD; UAS-aPKC$^{\Delta N}$, UAS-Ras$^{V12}$/+* |
| D-F | Tumor | *tj-Gal4, tubulin-Gal80$^{ts}$/UAS-GFP KD; UAS-aPKC$^{\Delta N}$, UAS-Ras$^{V12}$/+* |
| | Tumor + Tep3 KD | *tj-Gal4, tubulin-Gal80$^{ts}$/UAS-Tep3 KD; UAS-aPKC$^{\Delta N}$, UAS-Ras$^{V12}$/+* |
| G | Tumor | *Hml$^{P2A}$-LHG/+; tj-Gal4, tubulin-Gal80$^{ts}$/UAS-GFP KD; UAS-aPKC$^{\Delta N}$, UAS-Ras$^{V12}$/LexAop-3xnls::mScarlet3* |
| | Tumor + Tep3 KD | *Hml$^{P2A}$-LHG/+; tj-Gal4, tubulin-Gal80$^{ts}$/UAS-Tep3 KD; UAS-aPKC$^{\Delta N}$, UAS-Ras$^{V12}$/LexAop-3xnls::mScarlet3* |

**Table S1.** Experimental genotypes

**Table S2.**

| Key Resources and Reagents | | |
|---|---|---|
| **REAGENT or RESOURCE** | **SOURCE** | **IDENTIFIER** |
| **Antibodies** | | |
| Mouse NimC1; Dilution 1:100 | Istvan Ando | PMID: 17363253 |
| Rabbit cDcp1; Dilution 1:100 | Cell Signaling Technology | RRID: AB_9578 |
| Mouse MMP1; Dilution 1:100 | Hybridoma Bank | RRID: AB_579780 |
| Mouse MMP1; Dilution 1:100 | Hybridoma Bank | RRID: AB_579781 |
| Mouse MMP1; Dilution 1:100 | Hybridoma Bank | RRID: AB_579779 |
| Laminin B; Dilution 1:500 | Abcam | ab47650 |
| Mouse Flag M2; Dilution 1:100 | Sigma-Aldrich | F3165 |
| Alexa Fluor 488 anti-mouse; Dilution 1:200 | Invitrogen | A11001 |
| Alexa Fluor 647 anti-mouse; Dilution 1:100 | Invitrogen | A31571 |
| Alexa Fluor 488 anti-rabbit; Dilution 1:200 | Invitrogen | A11008 |
| Alexa Fluor 647 anti-rabbit; Dilution 1:100 | Invitrogen | A21244 |
| **Chemicals and materials** | | |
| TRITC-Phalloidin; Dilution 1:500 | Sigma-Aldrich | P1951 |
| DAPI; Dilution 1:5000 | ThermoFisher Scientific | D1306 |
| Paraformaldehyde 16% | Electron Microscopy Sciences | 15710 |
| Phosphate Buffered Solution | Sigma-Aldrich | P4417 |
| Triton-X | Sigma-Aldrich | T8787 |
| Bovine Serum Albumin | Sigma Aldrich | A3912 |
| SlowFade Diamond Antifade Mount | Invitrogen | S36963 |
| DHE | Invitrogen | D23107 |
| Casein-FITC | G-Biosciences | 786-320 |
| Schneider’s Drosophila Medium | Gibco | 21720-024 |
| Fetal bovine serum | Cytiva Life Sciences | SH30070.03 |
| Penicillin-Streptomycin | ThermoFisher Scientific | 15140-122 |
| Propidium Iodide | ThermoFisher Scientific | P3566 |
| Difco Agar | Becton Dickinson | 214010 |
| Grape Juice | Signature SELECT Juice Grape | N/A |
| Yeast | Fleischmann's Yeast | 2139 |
| 60mm Petri dish | Corning | 430166 |
| **Experimental models: Organisms/strains** | | |

| | | |
|---|---|---|
| *w1118* | Bloomington Drosophila Stock Center (BDSC) | 5905 |
| *Gla/CyO-TwiGFP* | Bilder lab stock | N/A |
| *tubulin-Gal80$^{ts}$* | BDSC | 7108 |
| *vkg-GFP [CC00791]* | Allan C. Spradling | PMID: 17194782 |
| *Tep3$^{sk2}$/CyO-Tb* | National Institute of Genetics | M2L-1890 |
| *Tep qΔ* | Bruno Lemaitre | PMID: 28874153 |
| *Tep3-Gal4/SM6a* | BDSC | 78968 |
| *en-Gal4, UAS-RFP/CyO* | BDSC | 30557 |
| *nub-Gal4, UAS-GFP* | Bilder lab stock | N/A |
| *tj-Gal4, tubulin-Gal80$^{ts}$* | Kyoto Drosophila Stock Center | 104055 |
| *tj-LHG, tubulin-Gal80$^{ts}$* | This paper | N/A |
| *mirr-Gal4/TM3* | Georg Halder | PMID: 19882738 |
| *Hml$^{P2A}$-Gal4* | Alf Herzig | PMID: 36093870 |
| *Hml$^{P2A}$-LHG* | This paper | N/A |
| *Pxn-LexA, LexAop-mCherry* | Jiwon Shim | PMID: 32082322 |
| *UAS-mCD8GFP* | BDSC | 32187 |
| *UAS-luciferase* | BDSC | 35788 |
| *UAS-aPKC$^{\Delta N}$* | BDSC | 51673 |
| *UAS-Ras$^{V12}$* | BDSC | 4847 |
| *UAS-rpr* | BDSC | 5824 |
| *UAS-hid:UAS-rpr* | Katja Bruckner | PMID: 31735669 |
| *UAS-p35* | BDSC | 5073 |
| *UAS-DIAP1* | BDSC | 6657 |
| *UAS-MMP1* | BDSC | 58700 |
| *UAS-Timp* | BDSC | 58708 |
| *UAS-Tep3-FLAG* | This paper | N/A |
| *UAS-GFP KD* | BDSC | 41552 |
| *UAS-mCherry KD* | BDSC | 35785 |
| *UAS-Tep3 KD* | BDSC | 56020 |
| *UAS-Tep3 KD* | BDSC | 63660 |
| *UAS-vkg KD* | Vienna Drosophila Resource Center | v106812 |
| *UAS-cg25c KD* | BDSC | 44520 |
| *UAS-lanB1 KD* | Vienna Drosophila Resource Center | 23119 |
| *UAS-lanB2 KD* | BDSC | 55388 |
| *UAS-MMP1 KD* | BDSC | 31489 |
| *UAS-Duox KD* | BDSC | 32903 |
| *LexAop-3xnls::mScarlet3* | This paper | N/A |
| *LexAop-aPKC$^{\Delta N}$-T2A-Ras$^{V12}$* | This paper | N/A |
| *LexAop-rpr* | Denise Montell | PMID: 33184261 |
| **Recombinant DNA** | | |

| | | |
|---|---|---|
| pUAST-attB vector | Drosophila Genomics Resource Center | 1419 |
| pJFRC19 | addgene | 26224 |
| pHML-P2A-Gal4 | Alf Herzig | PMID: 36093870 |
| pattB-LHG | Laura Mathies | N/A |
| Tep3 cDNA | Drosophila Genomics Resource Center | 4808 |
| **Software and algorithms** | | |
| FIJI | ImageJ | http://fiji.sc |
| Zeiss Imaging Software (Zen) | Zeiss | https://www.zeiss.com/microscopy/us/products/software/zeiss-zen.html |
| Graphpad | Prism | Ver 10 |

**Table S2.** Key Resources and Reagents
